# Circ-0075305 hinders gastric cancer stem cells by indirectly disrupting TCF4–β-catenin complex and downregulation of SOX9

**DOI:** 10.1038/s42003-024-06213-6

**Published:** 2024-05-07

**Authors:** Qi-Yue Chen, Kai-Xiang Xu, Xiao-Bo Huang, Deng-Hui Fan, Yu-Jing Chen, Yi-Fan Li, Qiang Huang, Zhi-Yu Liu, Hua-Long Zheng, Ze-Ning Huang, Ze-Hong Lin, Yu-Xiang Wang, Jun-Jie Yang, Qing Zhong, Chang-Ming Huang

**Affiliations:** 1 Department of Gastric Surgery, Fujian Medical University Union Hospital, Fuzhou, China; 2 Department of General Surgery, Fujian Medical University Union Hospital, Fuzhou, China; 3 Key Laboratory of Ministry of Education of Gastrointestinal Cancer, Fujian Medical University, Fuzhou, China

**Keywords:** Cancer stem cells, Gastric cancer

## Abstract

CircRNAs are covalently closed, single-stranded RNA that form continuous loops and play a crucial role in the initiation and progression of tumors. Cancer stem cells (CSCs) are indispensable for cancer development; however, the regulation of cancer stem cell-like properties in gastric cancer (GC) and its specific mechanism remain poorly understood. We elucidate the specific role of *Circ-0075305* in GC stem cell properties. *Circ-0075305* associated with chemotherapy resistance was identified by sequencing GC cells. Subsequent confirmation in both GC tissues and cell lines revealed that patients with high expression of *Circ-0075305* had significantly better overall survival (OS) rates than those with low expression, particularly when treated with postoperative adjuvant chemotherapy for GC. In vitro and in vivo experiments confirmed that overexpression of *Circ-0075305* can effectively reduce stem cell-like properties and enhance the sensitivity of GC cells to Oxaliplatin compared with the control group. *Circ-0075305* promotes RPRD1A expression by acting as a sponge for corresponding miRNAs. The addition of LF3 (a β-catenin/TCF4 interaction antagonist) confirmed that RPRD1A inhibited the formation of the TCF4–β-catenin transcription complex through competitive to β-catenin and suppressed the transcriptional activity of stem cell markers such as SOX9 via the Wnt/β-catenin signaling pathway. This leads to the downregulation of stem cell-like property-related markers in GC. This study revealed the underlying mechanisms that regulate *Circ-0075305* in GCSCs and suggests that its role in reducing β-catenin signaling may serve as a potential therapeutic candidate.

## Abstract (web summary)

Circular RNA Circ0075305 hinders gastric cancer stem cells by indirectly increasing the expression of RPRD1A. In turn, RPRD1A disrupts TCF4-β-catenin complex formation and promotes downregulation of SOX9 transcription.

## Introduction

CSCs are a distinct population of cells with the unique ability of self-renewal and differentiation. They contribute to tumor recurrence and metastasis owing to their remarkable resistance to chemotherapy^1–3^. Extensive evidence has confirmed the presence of CSCs in GC, which play a crucial role in GC initiation, progression, and drug resistance^4^. To develop efficacious therapeutic strategies targeting CSCs, it is imperative to understand the underlying regulatory mechanisms in greater depth^5^.

CircRNAs are a unique type of non-coding RNA formed by a covalent bond between 3′ and 5′ ends, creating a closed continuous loop. They are generated by back-splicing of precursor mRNA. Mounting evidence suggests that CircRNAs play an important role in the initiation and progression of various tumors, including GC^6,7^. Notably, CircRNAs regulate cancer stem cells through classical competing endogenous RNA mechanisms. Extensive research has focused on understanding the roles of CircRNAs in tumor stem cell-like properties and its involvement in chemotherapy resistance^8–10^. Multiple studies have demonstrated that sustained activation of the Wnt/β-catenin pathway confers self-renewal growth properties to cancer cells, which are associated with epithelial-mesenchymal transition (EMT), chemotherapy resistance, tumor immune regulation, and cancer stem cell-like properties^11–14^. Specifically, this pathway plays a crucial role in regulating cancer stem-like properties and chemoresistance by upregulating the mRNA expression of downstream target genes. For example, CircFAM73A influences the stem cell-like characteristics of GC by mediating β-catenin signaling^15^. However, the precise role of CircRNAs in β-catenin activation and stem cell-like properties of GC remains elusive.

In this study, our analysis focused on CircRNAs associated with chemotherapy resistance in GC. We identified *Circ-0075305* (Circ-MAML1) as a downregulated CircRNA in tumor tissues, suggesting its potential role as a molecule involved in drug resistance. Reduced expression of *Circ-0075305* is associated with poor prognosis GC and decreased sensitivity to chemotherapy among patients with GC. Interestingly, *Circ-0075305* was found to promote the expression of RPRD1A through sponge adsorption. In the nucleus, RPRD1A competitively binds to TCF4 and β-catenin and inhibits the Wnt/β-catenin signaling pathway. The downregulation of cancer stem cell-like markers such as CD44 and SOX9, which are associated with GC, inhibits drug resistance in GC^16–19^. These findings suggest that *Circ-0075305* may serve as a prognostic biomarker for patients undergoing postoperative chemotherapy for GC. This may provide original insights into the personalized diagnosis and treatment of GC, as well as targeted therapy against GCSCs.

## Results

### *Circ-0075305* is down-regulated in GC and associated with unfavorable prognosis

To identify CircRNAs associated with chemotherapy resistance in GC, we analyzed 45 genes that were differentially expressed between HGC-27 GC cell lines and their drug-resistant strains. *Circ-0075305* (Circ-MAML1) was significantly downregulated in the HGC-27 GC cell line compared to the human normal gastric epithelial cell line GES-1 (Supplementary Fig. 1a). The genomic location of *Circ-0075305* is chr5:179159850–179204287.

**Fig. 1:**
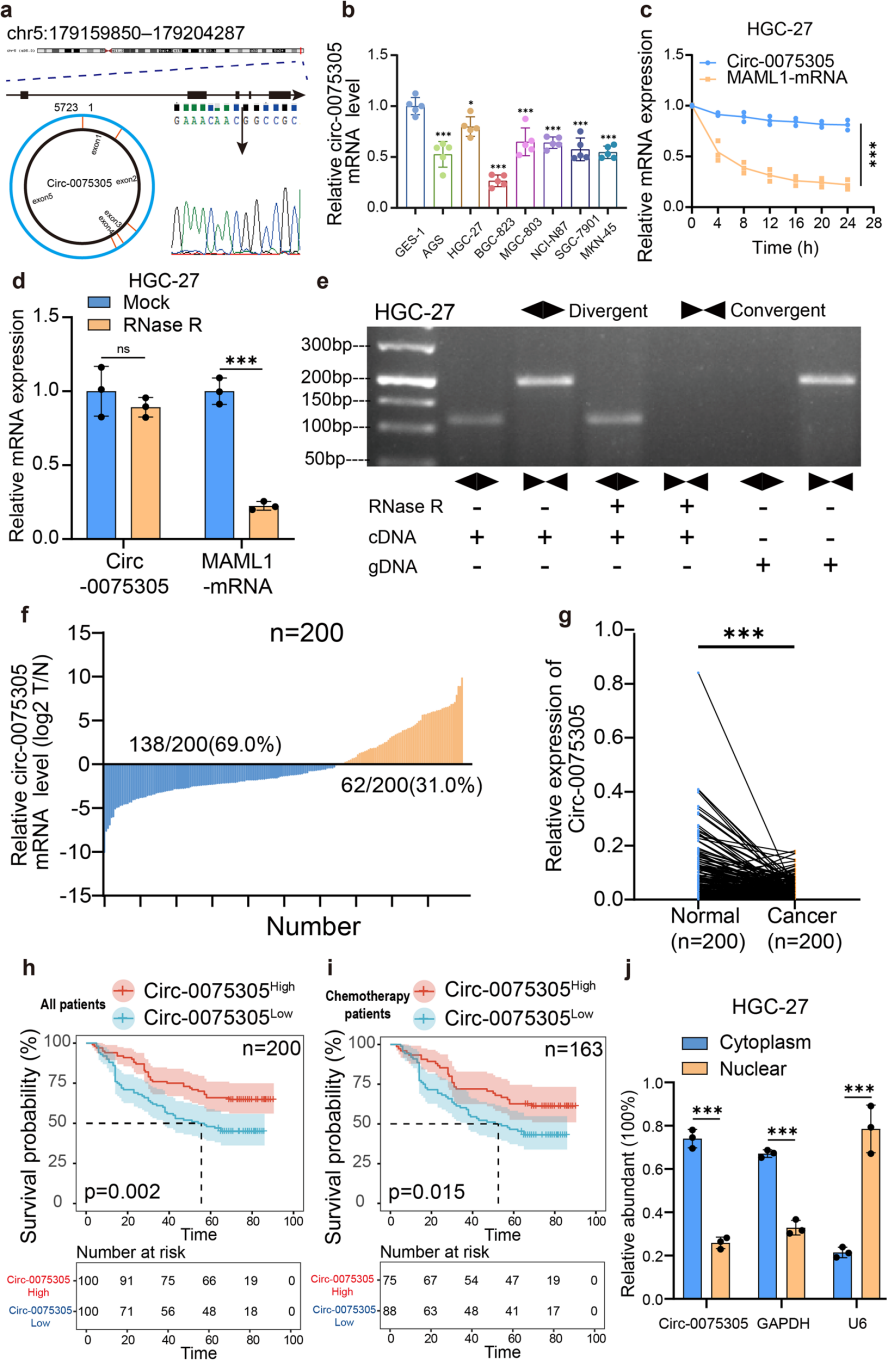
Circ-007530 significantly downregulated in GC. **a** *Circ-0075305* host gene chromosome location and Sanger sequencing verified the *Circ-0075305* back splicing structure. **b** Detection of *Circ-0075305* expression in gastric cancer cell lines using qRT-PCR. **c** Stability of CircRNA in HGC-27 cells was verified using an actinomycin D assay. **d** The characteristics of CircRNAs in HGC-27 cells that were not easily digested were verified using RNase R exonuclease digestion experiments. **e** The cyclic properties of CircRNAs in HGC-27 cells were verified by DNA gel electrophoresis. **f** The relative expression of *Circ-0075305* in 200 pairs of human gastric cancer tissues using RT-qPCR. **g** *Circ-0075305* expression and one-to-one pairing were detected using qRT-PCR. **h** Analysis of the impact of *Circ-0075305* expression on the OS rate of patients with GC. **i** Analysis of the impact of *Circ-0075305* expression on the OS rate in postoperative chemotherapy patients with GC. **j** The locations of CircRNAs were verified by performing nucleoplasmic separation experiments in HGC-27. Data are represented as the mean ± SD and analyzed by Student’s t-test or one-way analysis of variance (ANOVA). NS, no significance, *P < 0.05, **P < 0.01, ***P < 0.001 for groups connected by horizontal lines. *P*-values < 0.05 were considered statistically significant. **c**, **d**, **j**: *n* = 3 per group; **b**: *n* = 5 per group.

To confirm the characteristics of the back splicing g, specific primers targeting the *Circ-0075305* junction site were designed. We validated the head-to-tail back splicing in the RT – PCR product of *Circ-0075305* using Sanger sequencing (Fig. 1a).

qRT-PCR results showed the highest *Circ-0075305* abundance in HGC-27 cells and the lowest in BGC-823 cells (Fig. 1b). Therefore, we chose HGC-27 and BGC-823 cell lines for further investigation. The half-lives of *Circ-0075305* and MAML1 were determined in BGC-823 and HGC-27 cells using the transcription inhibitor actinomycin D. The results indicated that *Circ-0075305* was resistant to actinomycin D, while linear MAML1 transcription was inhibited (Fig. 1c and Supplementary Fig. 1b). Subsequent treatment of HGC-27 and BGC-823 cells with RNase R revealed that *Circ-0075305* was largely unaffected by digestion, whereas linear MAML1 transcription was inhibited. (Fig. 1d and Supplementary Fig. 1c).We amplified *Circ-0075305* using divergent primers and MAML1 mRNA using convergent primers. By utilizing cDNA and gDNA from both cell lines as templates, we observed that only cDNA produced an amplification product for *Circ-0075305* with divergent primers, while no product was obtained from gDNA (Fig. 1e and Supplementary Fig. 1e).

To investigate *Circ-0075305* expression in GC, we performed quantitative real-time PCR (qRT-PCR) and measured its levels in 200 pairs of tumor and adjacent normal tissues. The results indicated that *Circ-0075305* was significantly underexpressed (log2(T/N) < 0) in 69% (138/200) of tumor samples compared to normal tissues of the same patient (Fig. 1f, g).

Importantly, we determined by Kaplan-Meier analysis that patients with high *Circ-0075305* expression levels had a better OS rate than those with low expression levels, as seen in the postoperative chemotherapy group (Fig. 1h, i). Furthermore, statistical analysis of patient data demonstrated a significant correlation between elevated *Circ-0075305* expression and larger tumor size (*p* < 0.001), and advanced clinical stage (*p* = 0.010) (Table 1). Fluorescence in situ hybridization (FISH) was performed to elucidate the subcellular localization of *Circ-0075305* in GC cells. We observed that it was predominantly distributed in the cytoplasm (Supplementary Fig. 1f). Nuclear and plasma separation experiments corroborated these findings (Fig. 1j).

**Table. 1:** Correlation between the expression level of *Circ-0075305* in patients with gastric cancer and clinical data (*n* = 200)

Variable		*Circ-0075305*		
	Cases	Low	High	*p*-value
Age(years)				
<65	138	63	75	0.093*
≥65	62	37	25	
Gender				
Male	146	70	76	0.426
Female	54	30	24	
Size(cm)				
<5.0	139	53	86	<0.001***
≥5.0	61	47	14	
T Stages				
I	36	11	25	0.010*
II	20	7	13	
III	85	45	40	
IV	59	37	22	
N Stages				
I	58	22	36	0.042*
II	26	10	16	
III	49	29	20	
IV	67	39	28	
Chemotherapy				
No Accept	37	11	26	0.011*
Accept	163	89	74	

Note: *p < 0.05, ***p < 0.001 were considered statistically significant.

### *Circ-0075305* enhances the chemosensitivity of GC cells

**Fig. 2:**
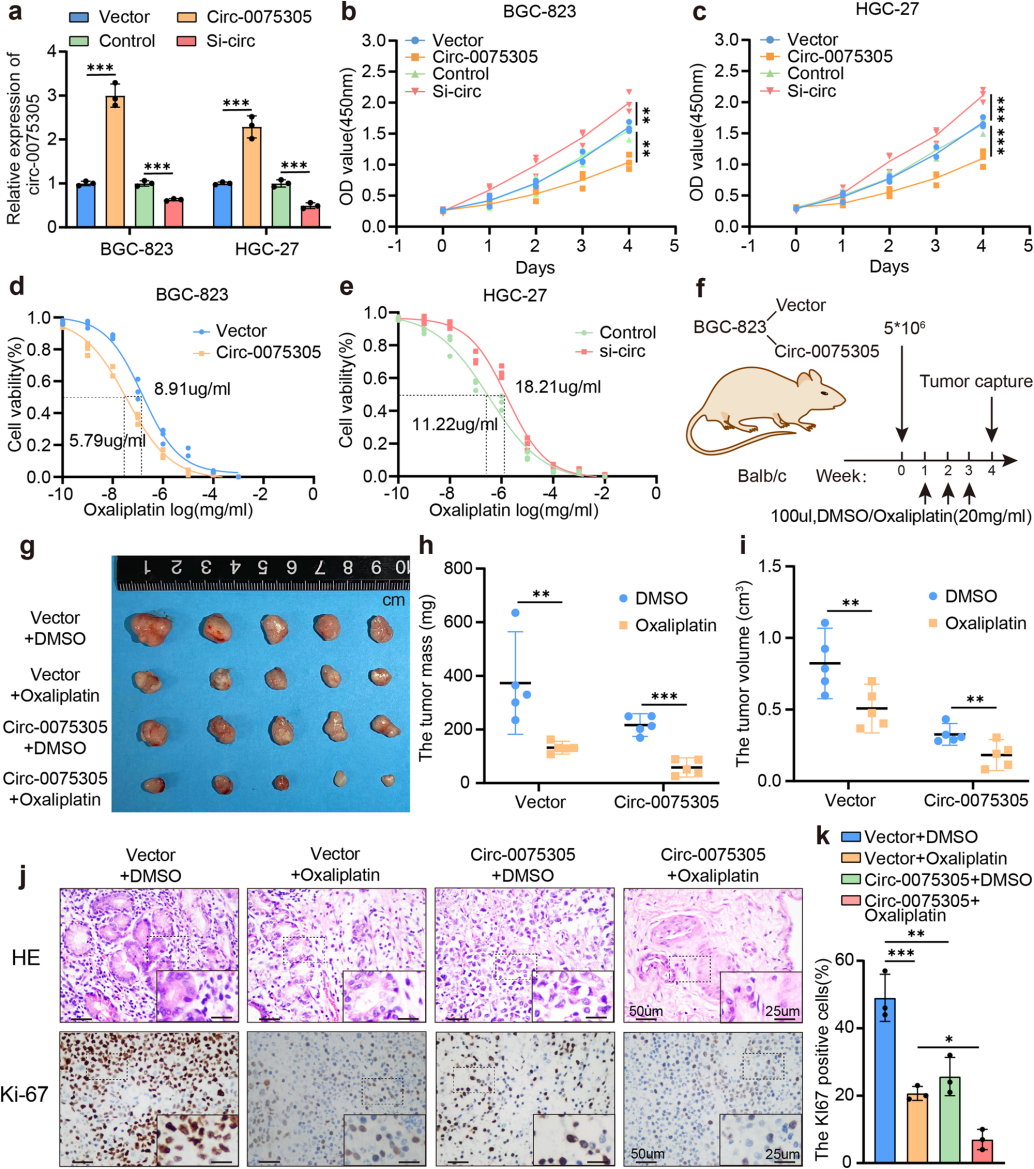
In vitro and in vivo investigation of the impact of Circ-0075305 up-regulation on chemotherapy sensitivity reduction in GC cells. **a** The transfection efficiency of overexpressed and knocked down *Circ-0075305* in gastric cancer cell lines was assessed using qRT-PCR. **b**, **c** The CCK8 assay was used to monitor the growth of BGC-823 and HGC-27 cells. **d**, **e** The CCK8 assay was used to monitor alterations in the sublethal concentration (IC50) of the chemotherapy drug (OXA) in the *Circ-0075305* GC cell line. **f** Schematic representation of nude mice treated with DMSO/OXA. **g** Subcutaneous tumor formation in mice inoculated with *Circ-0075305* GC cells after stable transfer of OXA/DMSO treatment. **h** The changes of tumor weight were recorded. **i** The changes of tumor volume were recorded. **j** The distribution of HE and Ki-67 expression in tumors was detected by using immunohistochemistry. **k** Statistical analysis of Ki-67 positive cells in tumor tissues. Data are represented as the mean ± SD and analyzed by Student’s t-test or one-way analysis of variance (ANOVA). *P < 0.05, **P < 0.01, ***P < 0.001 for groups connected by horizontal lines. *P*-values < 0.05 were considered statistically significant. **a**–**d**, **e**, **k**: *n* = 3 per group; **h**, **i**: *n* = 5 per group.

Overexpressing and silenced *Circ-0075305* GC cell lines were constructed to further investigate the impact of *Circ-0075305* on the biological behavior of GC cells (Fig. 2a). CCK-8 and transwell experiments demonstrated that *Circ-0075305* significantly suppressed proliferation and migration of GC cells (Fig. 2b, c and Supplementary Fig. 2a–f), which was consistent with its effect on subcutaneous xenograft tumors in nude mice (Supplementary Fig. 3a–h). We introduced the OXA concentration solvent into GC tumor spheres cultured to 50% confluence (Supplementary Fig. 4a). After OXA treatment, a significant reduction was observed in the survival rate of *Circ-0075305* overexpressed GC tumor spheres compared to the control group. Furthermore, the number of *Circ-0075305* overexpressed GC tumor spheres weakened by OXA significantly increased compared to that in the control group (Supplementary Fig. 4b–e). The number of organoids that exhibited overexpression or downregulation of *Circ-0075305* after OXA treatment was consistent with the aforementioned results (Supplementary Fig. 4f–h).

The CCK-8 assay was performed to detect changes in the semi-lethal concentrations (IC50) of chemotherapeutic drugs in GC cells. The results indicate that *Circ-0075305* overexpression significantly reduced the IC50 concentration of OXA in BGC-823 and HGC-27 cells (Fig. 2d, e). To validate our findings in vivo, a weekly injection of 100 µL OXA at a concentration of 20 mg/mL was administered in nude mice with seven days of subcutaneous tumor growth. The tumors were harvested in the fourth week (Fig. 2f). Consistent with the in vitro treatment, mice harbored *Circ-0075305* overexpressed cells treated with an equivalent dose of DMSO or OXA exhibited greater subcutaneous tumor regression compared to the control group (Fig. 2g–i). Simultaneously, we observed a significant reduction in the percentage of Ki-67 positive cells in mice harbored *Circ-0075305* overexpressed cells treated with an equivalent dose of DMSO or OXA (Fig. 2j–k).

### *Circ-0075305* suppressed the GC stem cell-like properties

Numerous studies have shown that tumor stem cell-like properties play a crucial role in determining the response of tumors to chemotherapy^20–22^. Therefore, we hypothesized that *Circ-0075305* may modulate the chemosensitivity of GC cells by regulating their stem cell-like properties. Overexpression of *Circ-0075305* significantly inhibited the proliferation of GC tumor spheres in vitro, whereas downregulation of *Circ-0075305* had the opposite effect (Supplementary Fig. 5a, b). In GC organoids, overexpression of *Circ-0075305* significantly inhibited the size of the organoids compared to that in the control group, whereas downregulation of *Circ-0075305* had the opposite effect (Supplementary Fig. 5c, d).

**Fig. 3:**
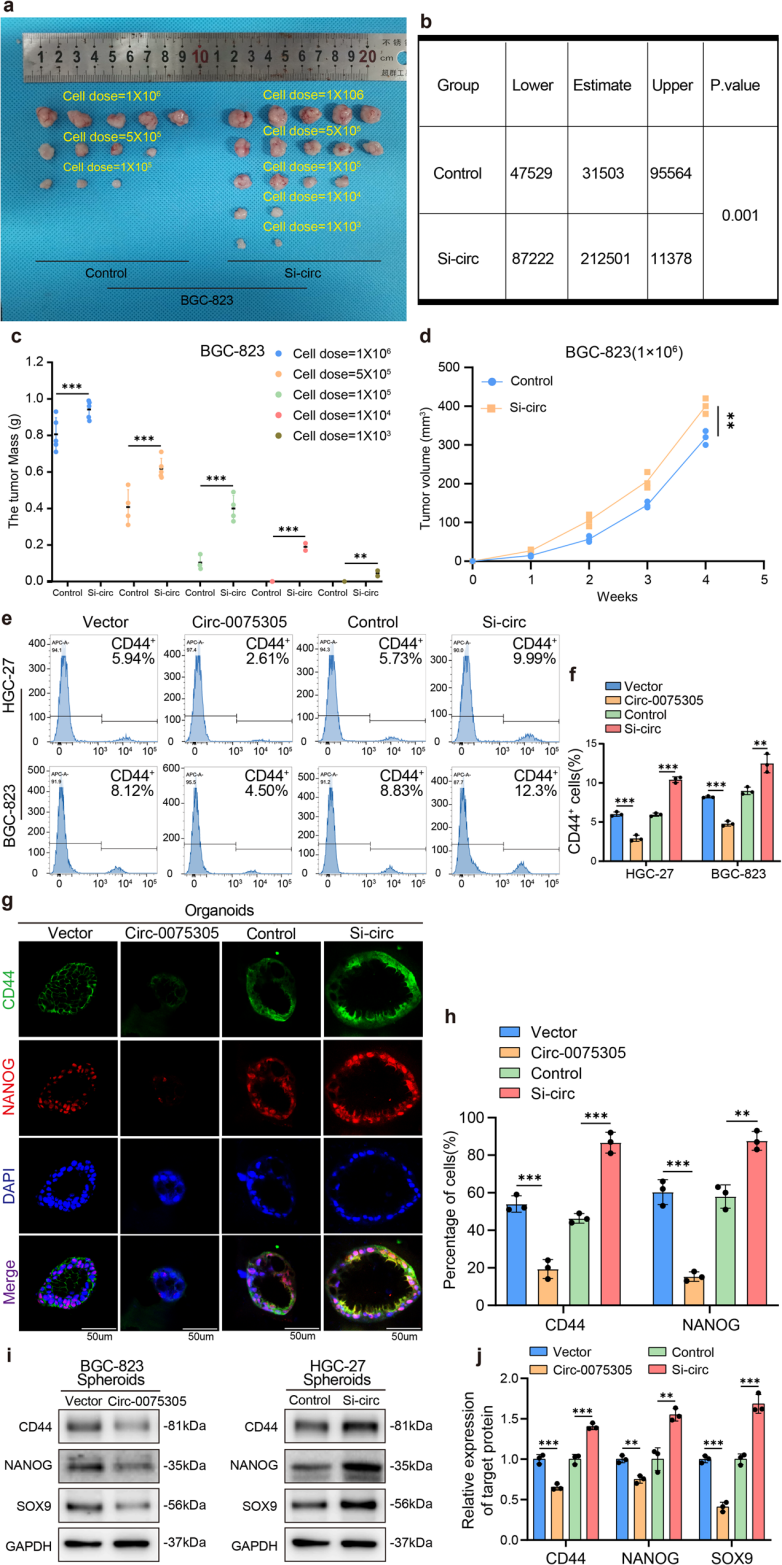
Up-regulation of Circ-0075305 can reduce stem-like properties of GC in vitro and in vivo. **a** Tumor formation frequencies for different numbers of the BGC-823 cells with or without *Circ-0075305* knockdown. **b** The dilution of **a** was statistically analyzed for tumor formation. **c** The changes of tumor weight were recorded. **d** The changes of tumor volume were recorded. **e** Flow cytometry reveals the percentage of CD44+ cells within the HGC-27 and BGC-823 cell populations, following transfection with either the control vector or *Circ-0075305* for overexpression or suppression. **f** Quantitative assessment of the flow cytometry results from the experiment conducted in **e**. **g** Immunofluorescence images of CD44 and NANOG were obtained from gastric cancer organoids transfected with a vector, *Circ-0075305* overexpression or *Circ-0075305* knockdown. **h** The proportions of CD44 and NANOG positive GC organoids in **g** were statistically analyzed. **i** Western blot was used to analyze the effect of *Circ-0075305* on gastric cancer stem cell-like characteristics. **j** Statistical interpretation of the results showcased in **i**. Data are represented as the mean ± SD and analyzed by Student’s t-test. **P < 0.01, ***P < 0.001 for groups connected by horizontal lines. *P*-values < 0.05 were considered statistically significant. **f**, **h**, **j**: *n* = 3 per group; **c**, **d**: *n* = 5 per group.

We further investigated the impact of *Circ-0075305* on the stem cell-like properties of GC cells in vivo. Nude mice were subcutaneously injected with *Circ-0075305*-downregulated and control BGC-823 GC cell line. Data on tumor formation rate and number in both groups were collected. Tumor formation rate and number exhibited a statistically significant increase in the *Circ-0075305*-downregulated group compared to the control group (Fig. 3a, b), Tumor weight and volume were also increased (Fig. 3c, d), indicating *Circ-0075305* inhibits robust tumor growth in vivo.

Numerous studies have shown that cancer cells with the CD44+ surface marker possess characteristics of tumor stem cells, such as self-renewal and tumor initiation^23–25^. Flow cytometry analysis revealed a significant decrease in the proportion of CD44+ positive cells in GC cell lines and tumor spheres in the *Circ-0075305* overexpression group compared to that in the control group (Fig. 3e, f and Supplementary Fig. 5e, f). Additionally, cell fluorescence experiments demonstrated that overexpression of *Circ-0075305* significantly inhibited the growth of GC tumor spheres and downregulated the expression of CD44 and NANOG compared to the control group. Moreover, knockdown of *Circ-0075305* notably facilitated the growth of GC tumor spheres and upregulated CD44 and NANOG expression (Supplementary Fig. 5i–l). The cell fluorescence assay conducted on GC organoids with overexpressed/knockdown *Circ-0075305* (Fig. 3g, h) confirmed the aforementioned findings.

Our experiment demonstrated a significant reduction in CD44- and NANOG-positive cells within the subcutaneous tumors of mice treated with OXA compared to the control group treated with an equivalent dose of DMSO. This effect was specifically observed in the *Circ-0075305* overexpression group (Supplementary Fig. 5m, n). Western blot analysis at the protein level revealed a significant downregulation of CD44, NANOG, and SOX9 expression levels in *Circ-0075305* overexpressing GC cell lines, whereas the opposite results were observed upon attenuation of *Circ-0075305* (Supplementary Fig. 5g, h). Furthermore, changes in the expression of stemness-related markers in GC tumor spheres following *Circ-0075305* overexpression/knockdown were consistent with the above findings (Fig. 3i, j).

### *Circ-0075305* adsorbs downstream targets to inhibit its transcriptional activity

**Fig. 4:**
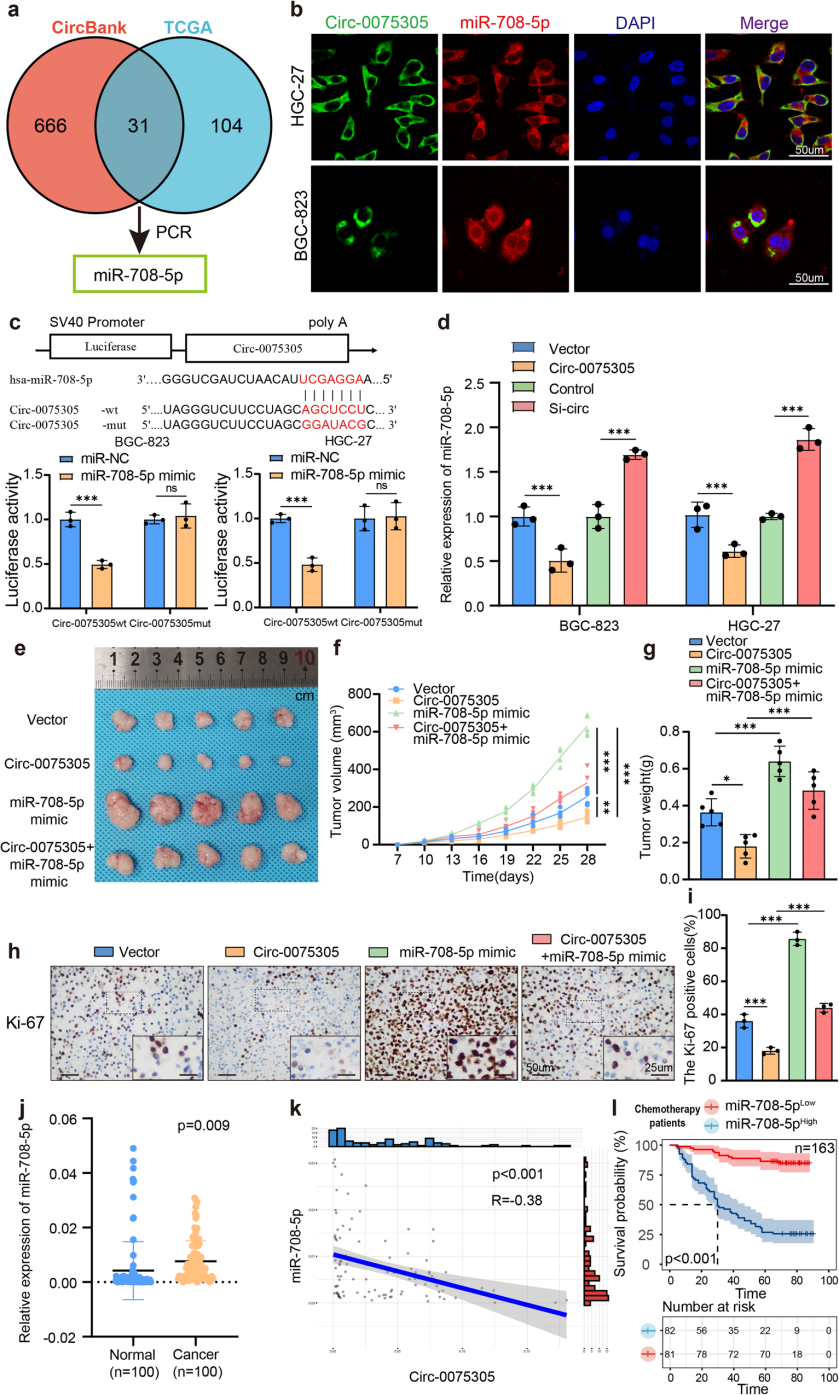
Circ-0075305 targets and inhibits miR-708-5p activity. **a** Multiple databases predicted miRNAs with binding sites for *Circ-0075305* and screened for *miR-708-5p* in *Circ-0075305* GC cell lines with stable expression and silencing by qRT-PCR. **b** The co-location of *Circ-0075305* (green) and *miR-708-5p* (red) in the cytoplasm of GC cells was verified by FISH. Cell nucleuses were counterstained with DAPI (blue). **c** Dual-luciferase assay was conducted to confirm the structural and functional binding relationship between *Circ-0075305* and *miR-708-5p*. **d** Changes in *miR-708-5p* in *Circ-0075305* GC cell lines were detected using qRT-PCR. **e** Tumor formation in GC cells inoculated subcutaneously into mice. **f** The changes of tumor volume were recorded. **g** The changes of tumor weight were recorded. **h** The expression and distribution of Ki-67 in these tumors were detected using immunohistochemistry. **i** Statistical analysis of Ki-67 positive cells in tumor tissues. **j** Expression levels of *miR-708-5p* in GC tissues and adjacent normal tissues were quantified using qRT-PCR. **k** The association between *Circ-0075305* and *miR-708-5p* expression levels in GC tissues was investigated using qRT-PCR. **l** Analysis of the impact of *miR-708-5p* expression on the OS rate in postoperative chemotherapy patients with GC. Data are represented as the mean ± SD and analyzed by Student’s t-test or one-way analysis of variance (ANOVA). NS, no significance, *P < 0.05, ***P < 0.001 for groups connected by horizontal lines. *P*-values < 0.05 were considered statistically significant. **c**, **d**, **i**: *n* = 3 per group; **f**, **g**: *n* = 5 per group.

CircRNAs, mainly expressed in the cytoplasm, regulate downstream transcription by targeting miRNAs and modulating stem cell-like properties of GC cells^26,27^. To identify downstream miRNAs with potential binding affinities, 31 miRNAs (including *miR-708-5p*) were obtained via joint prediction from the CircBank and TCGA databases (Fig. 4a and Supplementary Fig. 6d). To further identify downstream miRNA-binding partners, qRT-PCR was performed to detect differences in miRNA expression in GC cell lines overexpressing/knocking down *Circ-0075305*. miR-302a-3p and *miR-708-5p* were identified as potential binding partners (Supplementary Fig. 6a, b). qRT-PCR confirmed no significant differences in miR-302a-3p expression levels between GC and normal tissues (Supplementary Fig. 6c). However, *miR-708-5p* expression was significantly higher in GC tissues than in normal tissues (Fig. 4j).

FISH revealed the co-localization and enrichment of *Circ-0075305* and *miR-708-5p* within the cytoplasm, confirming their spatial relationship during the interaction (Fig. 4b). We generated two luciferase reporter plasmids for *Circ-0075305*: Two luciferase reporter constructs, one containing a wild-type *miR-708-5p* binding site, and the other harboring a mutant site, were utilized. Our findings demonstrated that ectopic expression of *miR-708-5p* significantly repressed the activity of the wild-type luciferase reporter, whereas no significant alteration was observed in its mutant counterpart (Fig. 4c). The expression of *miR-708-5p* was significantly downregulated in GC cell lines overexpressing *Circ-0075305* compared to that in the control group. (Fig. 4d).

We investigated the impact of *Circ-0075305* and *miR-708-5p* on GC occurrence and progression in vivo. Nude mice were subcutaneously injected with equal amounts (1 × 10^6^) of BGC-823 overexpressing *Circ-0075305* and transiently mimicking *miR-708-5p*. After 4 weeks, the collected subcutaneous tumors were analyzed (Fig. 4e). Compared to the control group, the overexpressed *Circ-0075305* group exhibited a significantly lower volume and weight. Conversely, the transient *miR-708-5p* mimic group showed a significantly higher volume and weight than the control group, which was reversed by co-transfection with *Circ-0075305* and the *miR-708-5p* mimic (Fig. 4f, g). Immunohistochemistry analysis showed a significant decrease in Ki-67 positive cells in the *Circ-0075305* group compared to the control group. Conversely, there was a significant increase in Ki-67 positive cells observed in the transient *miR-708-5p* mimic group compared to controls, which was effectively reversed by co-transfection with both *Circ-0075305* and the *miR-708-5p* mimic (Fig. 4h, i).

Meanwhile, a negative correlation was observed between the expression of *Circ-0075305* and *miR-708-5p* in GC tissues (Fig. 4k), Furthermore, clinical data analysis of the postoperative chemotherapy group of GC patients revealed that those with high expression levels of *miR-708-5p* exhibited a significantly worse OS rate than those with low expression levels (Fig. 4l).

### *Circ-0075305* modulates the expression of RPRD1A by enhance its transcription

**Fig. 5:**
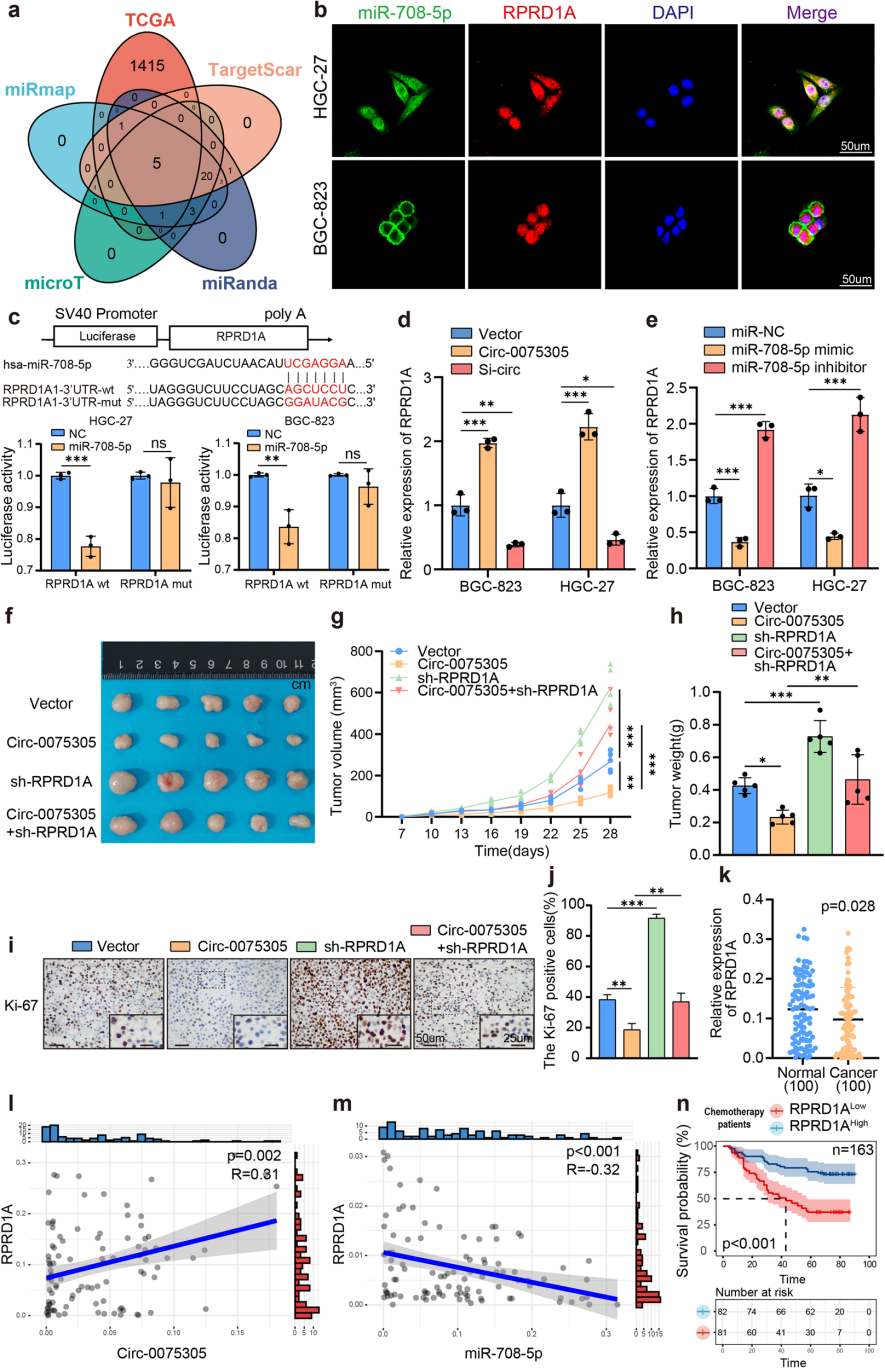
Mechanism of interaction between miR-708-5p and RPRD1A mRNA. **a** Multiple databases predicted mRNAs with binding sites for *miR-708-5p* and screened for RPRD1A in *miR-708-5p* mimics/inhibitors in with stable expression and silencing by qRT-PCR. **b** The co-location of *miR-708-5p* (green) and RPRD1A (red) in the cytoplasm of GC cells was verified by FISH. Cell nuclei were counterstained with DAPI (blue). **c** Dual-luciferase reporter assay was conducted to confirm the structural and functional binding relationships between *miR-708-5p* and RPRD1A. **d** qRT-PCR was used to assess the expression of RPRD1A in GC cell lines with stable overexpression or knockdown of *Circ-0075305*. **e** The expression level of RPRD1A was quantified by qRT-PCR in GC cell lines transfected with either the *miR-708-5p* mimic or inhibitor. **f** Tumor formation in GC cells inoculated subcutaneously into mice. **g** The changes of tumor volume were recorded. **h** The changes of tumor weight were recorded. **i** The expression and distribution of Ki-67 in these tumors were detected using immunohistochemistry. **j** Statistical analysis of Ki-67 positive cells in tumor tissues. **k** Expression levels of RPRD1A in GC tissues and adjacent normal tissues were quantified using qRT-PCR. **l** The association between *Circ-0075305* and RPRD1A expression levels in GC tissues was investigated using qRT-PCR. **m** The association between *miR-708-5p* and RPRD1A expression levels in GC tissues was investigated using qRT-PCR. **n** Analysis of the impact of RPRD1A expression on the OS rate in postoperative chemotherapy patients with GC. Data are represented as the mean ± SD and analyzed by Student’s t-test or one-way analysis of variance (ANOVA). NS, no significance, *P < 0.05, **P < 0.01, ***P < 0.001 for groups connected by horizontal lines. *P*-values < 0.05 were considered statistically significant. **c**, **d**, **e**, **j**: *n* = 3 per group; **g**, **h**: *n* = 5 per group.

The classical ceRNA mechanism involves the interplay between CircRNAs, miRNAs, and mRNAs. To comprehensively predict the downstream target genes of *miR-708-5p*, we utilized various tools, such as miRDB, TCGA, TargetScan, and miRanda, to identify mRNA (FOXJ3, NRAS, RARG, MAP3K3, and RPRD1A) that bind to *miR-708-5p* (Fig. 5a). To further elucidate the downstream mRNA-binding partners, we performed qRT-PCR analysis of mRNA expression in GC cell lines transfected with *Circ-0075305* overexpression/knockout and transient *miR-708-5p* mimic/inhibitor. Using this approach, we identified RPRD1A as a potential binding partner (Supplementary Fig. 7a, b).

To investigate the expression level of RPRD1A in GC tissues, we analyzed RPRD1A expression using the mouse GC (GSE35808 and GES93173) and human GC (GSE26942 and GES29272) datasets available in the GEO database. Our findings demonstrated that RPRD1A exhibited consistently low expression levels in GC tissues, suggesting a potential inhibitory effect on the development and progression of this disease (Supplementary Fig. 8a, b). Concurrently, analysis of histone samples from multiple patient groups within our center revealed a downregulation of RPRD1A expression in GC tissues and upregulation in normal tissues, thereby validating the aforementioned findings (Supplementary Fig. 8c, d).

FISH revealed the subcellular localization of *miR-708-5p* and RPRD1A, with cytoplasmic and nuclear enrichment, respectively (Fig. 5b), clearly defining their positional relationship. To investigate the mechanism underlying the interaction between *miR-708-5p* and RPRD1A, we confirmed their structural and functional binding using double-luciferase reporter assays (Fig. 5c). Upregulation of RPRD1A was observed in GC cell lines that overexpressed *Circ-0075305*, whereas its downregulation occurred upon transfection with the *miR-708-5p* mimic. In contrast, immediate transfection with the *miR-708-5p* inhibitor produced opposing outcomes (Fig. 5d, e), indicating mutual promotion between *Circ-0075305* and RPRD1A.

We further investigated the impact of *Circ-0075305* and RPRD1A on the occurrence and progression of GC in vivo. An equal number (1 × 10^6^) of overexpressed *Circ-0075305* and RPRD1A knockdown BGC-823 cells were injected subcutaneously into nude mice, and tumor samples were collected after 4 weeks (Fig. 5f). The overexpressed *Circ-0075305* group exhibited a significant reduction in both volume and weight compared to the control group, while the knockdown RPRD1A group showed a significant increase in both parameters. However, co-transfection with *Circ-0075305* and sh-RPRD1A reversed these effects (Fig. 5g, h). Immunohistochemistry analysis revealed a significant decrease in the proportion of Ki-67 positive cells in the *Circ-0075305* group compared to that in the control group. Conversely, there was a marked increase in the percentage of Ki-67 positive cells in the RPRD1A knockdown group, which was rescued by co-transfection with *Circ-0075305* and sh-RPRD1A (Fig. 5i, j).

RPRD1A expression was significantly lower in GC tissues than that in normal tissues, as confirmed by qRT-PCR (Fig. 5k). This suggests that RPRD1A functions as a tumor suppressor and exerts similar effects on *Circ-0075305* in impeding the progression of GC. Furthermore, a positive correlation was observed between *Circ-0075305* and RPRD1A mRNA expression in GC tissues (Fig. 5l). The expression levels of *miR-708-5p* and RPRD1A mRNA in GC tissues showed a significant inverse correlation (Fig. 5m).

Analysis of clinical data of GC patients who underwent postoperative chemotherapy revealed that those with high RPRD1A expression exhibited a significantly improved OS rate compared to their low-expression counterparts (Fig. 5n).

### *Circ-0075305* coordinates downstream molecules to jointly regulate the stem-like properties of GC

Previous experiments have confirmed that *Circ-0075305* can attenuate the stem-like properties of GC and enhance the sensitivity of this malignancy to chemotherapy drugs. Given their close association, we hypothesized that *miR-708-5p* and RPRD1A modulate GC chemosensitivity of GC. Through GSEA analysis of TCGA, we identified a close association between *miR-708-5p* and RPRD1A and the proliferation and differentiation of GC stem cells (Supplementary Fig. 9a and Supplementary Fig. 10a). Furthermore, the differential expression patterns of *miR-708-5p* and RPRD1A were closely associated with the proliferation and differentiation of GC stem cells (Supplementary Fig. 10b).

The analysis of TCGA and ACRG datasets revealed that the high expression group of *miR-708-5p* significantly enhanced the IC50 value of OXA (Supplementary Fig. 9b, c). In the ACRG dataset analysis, the high expression group of RPRD1A notably reduced the IC50 value of OXA (Supplementary Fig. 10c). Alterations in the Wnt/β-catenin signaling pathway, whether through activation or inhibition, are frequently accompanied by changes in tumor stem-cell like properties and chemotherapy sensitivity^28–30^. In addition, the GSEA analysis demonstrated a positive association between elevated expression of *miR-708-5p* and activation of the Wnt/β-catenin signaling pathway (Supplementary Fig. 9d), while high expression of RPRD1A was found to be negatively correlated with Wnt/β-catenin signaling pathway activation (Supplementary Fig. 10d).

**Fig. 6:**
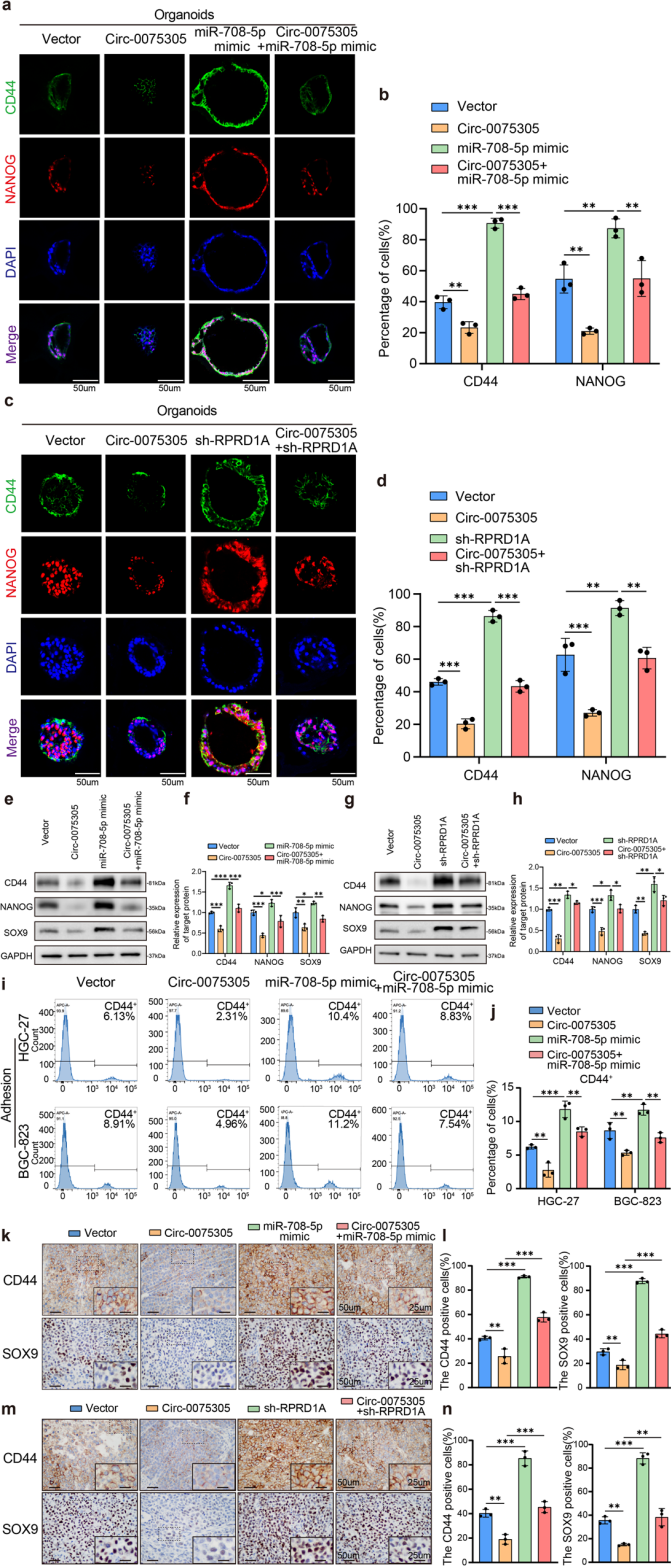
Circ-0075305/miR-708-5p/RPRD1A co-regulated stem cell-like properties of GC. **a** Immunofluorescence images of CD44 and NANOG were obtained from gastric cancer organoids transfected with a vector, *Circ-0075305* overexpression, *miR-708-5p* mimic expression, or both *Circ-0075305* overexpression and *miR-708-5p* mimic expression. **b** The proportions of CD44 and NANOG positive GC organoids in **a** were statistically analyzed. **c** Immunofluorescence images of CD44 and NANOG were obtained from gastric cancer organoids transfected with a vector, *Circ-0075305* overexpression, RPRD1A knockdown, or both. **d** The proportions of CD44 and NANOG positive GC organoids in Fig. c were statistically analyzed. **e** Western blot analysis was employed to detect alterations in stem cell-like property marker expression in GC cells transfected with *Circ-0075305* or/and *miR-708-5p* mimic. **f** Quantitative interpretation of the Western blot results from **e**. **g** Western blot analysis of stem cell-like property marker expression in GC cells transfected with *Circ-0075305* or/and sh-RPRD1A. **h** Quantitative interpretation of the Western blot results from **g**. **i** Proportion of CD44+ cells within HGC-27 and BGC-823 cell populations, transfected with *Circ-0075305* or/and *miR-708-5p* mimic, identified via flow cytometry. **j** Statistical analysis of the results in **i**. **k** Immunohistochemical detection of CD44 and SOX9 expression in tumors samples. **l** Quantitative assessment and correlation analysis of the indicators observed in **k**. **m** Immunohistochemical detection of the distribution of CD44 and SOX9 expression in tumors samples. **n** Statistical analysis of the relationships between the expression of the indicators in **m**. Data are represented as the mean ± SD and analyzed by one-way analysis of variance (ANOVA). *P < 0.05, **P < 0.01, ***P < 0.001 for groups connected by horizontal lines. *P*-values < 0.05 were considered statistically significant. **b**, **d**, **f**, **h**, **j**, **i**, **n**: *n* = 3 per group; **b**: *n* = 5 per group.

The effect of the *Circ-0075305*/*miR-708-5p*/RPRD1A axis on stem cell-like properties in GC was further validated through response experiments. Overexpression of *Circ-0075305* and transient transfection with an *miR-708-5p* mimic/inhibitor were performed in GC cell lines, and 3D tumor pellet formation experiments verified that *miR-708-5p* could reverse the effect of *Circ-0075305* in reducing stem-like properties (Supplementary Fig. 9e, g). sh-RPRD1A was observed to counteract the inhibitory effect of *Circ-0075305* on stem-like properties in GC, as demonstrated by in vitro experiments (Supplementary Fig. 9f, h and Supplementary Fig. 10i, l). Furthermore, a cell fluorescence recovery assay revealed that transfection with *Circ-0075305* or *miR-708-5p* mimic led to changes in the gastric cancer stem cell-related markers CD44 and NANOG; however, these effects were reversed by sh-RPRD1A. The function of *Circ-0075305* in reducing the aforementioned markers was counteracted by *miR-708-5p* and sh-RPRD1A (Supplementary Fig. 9i–l and Supplementary Fig. 10m–p). These findings were further validated in GC organoids transfected with overexpressed *Circ-0075305*, *miR-708-5p* mimic, and sh-RPRD1A (Fig. 6a–d).

GC cell lines expressing *Circ-0075305* and transient *miR-708-5p* mimics/inhibitors were constructed. Western blot analysis revealed that co-transfection of *Circ-0075305* and *miR-708-5p* mimic could reverse the upregulation or downregulation of RPRD1A expression induced by *miR-708-5p* mimic/inhibitor (Supplementary Fig. 10e, g). The alterations in the overexpression/knockdown group of *Circ-0075305* were consistent with those mentioned above (Supplementary Fig. 10f, h). Western blot analysis revealed that *Circ-0075305* downregulated the protein expression of CD44, NANOG, and SOX9, and this was rescued by *miR-708-5p* and sh-RPRD1A (Fig. 6e–h).

Flow cytometry analysis revealed that overexpression of *Circ-0075305* and transient *miR-708-5p* mimic in GC cell lines led to a reduction in the proportion of the CD44+ population within the overexpressing group. Conversely, the transient *miR-708-5p* mimic group exhibited an increase in CD44+ cells, which was reversed by co-transfection with *Circ-0075305* and *miR-708-5p* mimic (Fig. 6i–j). Moreover, flow cytometry analysis revealed that the knockdown of RPRD1A in gastric cancer cell lines resulted in marked elevated proportion of CD44+ cells (Supplementary Fig. 11a, b). The immunohistochemical findings of CD44 and NANOG were in accordance with the aforementioned experimental results (Fig. 6k–n).

### RPRD1A inhibits the formation of TCF4-β-catenin transcription complex and the stem cell-like characteristics of GC

**Fig. 7:**
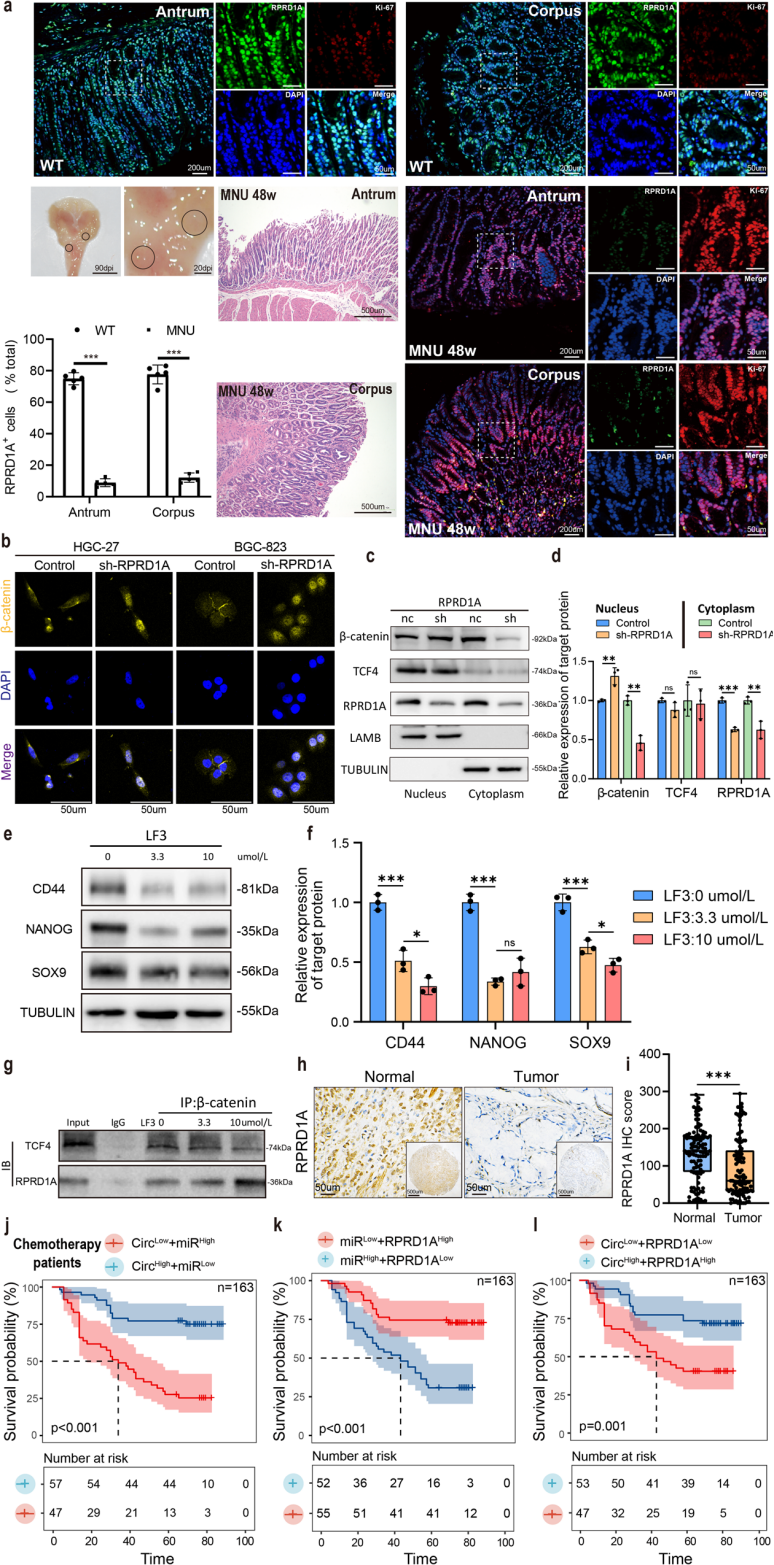
Circ-0075305 hinders the stem-like properties of GC by competitively binding with β-catenin through the RPRD1A axis. **a** Wild-type C57BL6 mice developed GAC at 48 weeks after MNU induction. The proportion of RPRD1A+ cells in MNU-induced mouse tumors was significantly lower than that in normal gastric tissue (body:74.3 ± 3.8%vs.9.1 ± 1.9%, *P* < 0.001; Sinus:76.9 ± 5.2%vs.10.2 ± 1.7%, *P* < 0.001). **b** Immunofluorescence was used to determine whether *Circ-0075305* affected the nucleation of β-catenin in GC cell lines. **c** The nucleocytoplasmic separation experiment was utilized to gauge the consequences of RPRD1A knockdown on TCF4 and β-catenin expression in nucleus and cytoplasm. **d** Statistical evaluation of results presented in **c**. **e** Western blot analysis was performed to assess alterations in the expression levels of CD44, NANOG, and SOX9 after the addition of different concentrations of the TCF4 antagonist (LF3) to HGC-27 cells. **f** Statistical evaluation of results presented in **e**. **g** Expression levels of β-catenin-bound RPRD1A and TCF4 in GC cell lines treated with varying concentrations of the TCF4 antagonist (LF3) were detected by CO-IP. **h** Immunohistochemical representation of RPRD1A in GC and normal tissue. **i** The box plot depicted the distributions of the immunohistochemical score of RPRD1A expression in gastric and normal tissues. The lower and upper sides of the box are the lower and upper quartiles. The whiskers are the two lines outside the box, that go from the minimum to the lower quartile and then from the upper quartile to the maximum. Each dot indicated score of individual patients. **j** Clinical data analysis was conducted to investigate the impact of *Circ-0075305* and *miR-708-5p* expression on the overall survival of patients with GC who received postoperative adjuvant chemotherapy. **k** Examination of *miR-708-5p* and RPRD1A’s impact on the overall survival of GC patients receiving postoperative adjuvant chemotherapy, based on patient clinical data. **l** The influence of *Circ-0075305* and RPRD1A expression on the overall survival of GC patient. Data are represented as the mean ± SD and analyzed by Student’s t-test or one-way analysis of variance (ANOVA). *NS* no significance, *P < 0.05, **P < 0.01, ***P < 0.001 for groups connected by horizontal lines. *P*-values < 0.05 were considered statistically significant. **d**, **f**: *n* = 3 per group; **a**: *n* = 5 per group.

To further investigate the expression of RPRD1A in the stomachs of mice, wild-type C57BL6 mice were induced with N-Nitroso-N-methylurea (MNU), and GAC developed after 48 weeks. Immunofluorescence and hematoxylin and eosin (HE) staining revealed a significant reduction in the proportion of RPRD1A+ cells in MNU-induced mouse tumors compared to that in normal stomach tissue (Fig. 7a).

Several preliminary studies have already demonstrated RPRD1A acts as a tumor suppressor by competitively binding with β-catenin and inhibiting the formation of TCF4–β-catenin transcription complex^31–33^. Our study, utilizing cell fluorescence in HGG-27 and BGC-823 cells with stable knockdown of RPRD1A, revealed an increase in the nuclear import of β-catenin, indicating that RPRD1A can effectively inhibit the nuclear expression of β-catenin (Fig. 7b). Meanwhile, a nucleocytoplasmic separation experiment assay was conducted on the established GC cell line (HGC-27) with RPRD1A knockdown, revealing that RPRD1A modulates the nuclear expression level of β-catenin while having no impact on the nuclear expression level of TCF4 (Fig. 7c, d). Therefore, it is suspected that RPRD1A does not affect protein expression by competitively binding to key molecules in the Wnt signaling pathway.

β-catenin activates the Wnt/β-catenin signaling pathway by binding to TCF4, thereby promoting the expression of stemness markers: CD44, NANOG, and SOX9. We treated HGC-27 cells with varying concentrations of the TCF4 antagonist LF3 and observed a dose-dependent decrease in the expression of CD44, NANOG, and SOX9 by performing Western blot analysis. These findings confirm the inhibitory effect of LF3 on stemness-associated markers (Fig. 7e, f). Furthermore, through the use of varying concentrations of LF3 in HGC-27, CO-IP analysis revealed a positive correlation between increasing LF3 concentration and enhanced binding of β-catenin to RPRD1A, as well as a significant decrease in β-catenin’s interaction with TCF4. This resulted in the reduced expression of the TCF4-β-catenin transcription complex (Fig. 7g).

To confirm the pivotal role of RPRD1A in repressing the expression of SOX9 and CD44 in GC, we initially assessed the expression of RPRD1A using immunohistochemistry in a microarray of GC patients with GC from our institution. Our findings indicated that RPRD1A was expressed at significantly lower levels in GC tissues than in adjacent normal tissues (Fig. 7h, i). Furthermore, correlation analysis revealed an inverse association between RPRD1A and CD44 (Supplementary Fig. 12a, b).

Further grouping analysis revealed that the *Circ-0075305* group with high expression and low expression of *miR-708-5p* exhibited a significantly higher OS rate following postoperative chemotherapy than the *Circ-0075305* group with low expression of *Circ-0075305* and high expression of *miR-708-5p* (Fig. 7j). The survival rate of patients with low *miR-708-5p* expression and high RPRD1A expression was higher than that of patients with high *miR-708-5p* expression and low RPRD1A expression (Fig. 7k). Moreover, the cohort with diminished levels of *Circ-0075305* and RPRD1A expression exhibited a reduced OS rate following postoperative chemotherapy compared with their counterparts with elevated *Circ-0075305* and RPRD1A expression (Fig. 7l).

## Discussion

Despite advancements in postoperative chemotherapy for cancer treatment, drug resistance resulting from the stem-like properties of certain GC cells remains the primary cause of mortality. To tackle this challenge, we focused on CircRNAs and their role in stem cell characteristics and drug resistance in GC. The analysis of clinical samples in our center revealed a significant correlation between high expression levels of *Circ-0075305* and improved OS rates in GC patients, as opposed to those with low expression levels. In vivo and in vitro experiments confirmed that *Circ-0075305* promoted the expression of RPRD1A by competitively binding to β-catenin with TCF4 through *miR-708-5p*. Inhibition of the Wnt/β-catenin signaling pathway can lead to down-regulation of GC stem cell-like markers, including CD44 and SOX9, thereby suppressing resistance characteristics in GC stem cells.

Several studies have demonstrated the pivotal role of CircRNAs in cancer progression and their potential impact on the efficacy of chemotherapy. The combination of hsa_circ_0003222 regulation and immunotherapy has been shown to effectively improve patient prognosis in small cell lung cancer^34^. CircPVT1 is significantly overexpressed in chemoresistant cell lines and is characterized by its ability to regulate sensitivity to chemotherapeutic drugs^35^. In the present study, we used qRT-PCR to quantify the expression level of *Circ-0075305* in GC tissues obtained from patients who received postoperative chemotherapy. Our findings suggest that the expression of *Circ-0075305* is significantly downregulated in GC tissues compared to that in their respective control groups. In contrast, patients with low *Circ-0075305* expression showed significantly poorer OS rates than those with high expression. Functional assays showed that overexpressing *Circ-0075305* reduced GC cell proliferation and migration. (Supplementary Fig. 2) while reducing their stem-like properties and enhancing chemosensitivity. CircRNAs primarily function as miRNA sponges to regulate downstream mRNA mechanisms. For instance, CircIFNGR2 indirectly targets KRAS by sequestering MiR-30b and inducing resistance to chemotherapeutic drugs^36^.

Bioinformatic prediction analysis and dual-luciferase reporter experiments confirmed that *Circ-0075305* can target and adsorb *miR-708-5p*. Additionally, TCGA database analysis revealed that the expression of *miR-708-5p* is lower in GC tissues than in normal tissues. This miRNA is downregulated in various cancer types. For example, MINCR uses *miR-708-5p* to upregulate CTNNB1 and activate the Wnt/β-catenin pathway, thereby promoting colon cancer development^37^. Furthermore, the lncRNA LOXL1-AS1 promotes GC tumorigenesis and stem cell differentiation by regulating the *miR-708-5p*/USF1 pathway^38^.

Next, using bioinformatics prediction and a dual-luciferase reporter assay, we identified the downstream target genes to which *miR-708-5p* binds. Our findings revealed that it specifically targets the 3’UTR of RPRD1A. We demonstrated the precise mechanism by which *Circ-0075305* regulates RPRD1A expression. RPRD1A plays different roles in different cancers, may enhance nuclear translocation of NRF2, thereby inducing the expression of genes that resist oxidative stress, maintain cancer cell survival and promote HCC development^39^. Meanwhile RPRD1A plays a crucial role in tumor stem cell formation, metastasis, and drug resistance^40^. Several studies have demonstrated the indispensability of RPRD1A dimerization in its inhibitory effect on Wnt signaling^41^.

Through multiple bioinformatics analyses, we discovered that RPRD1A has the potential to decrease the IC50 of OXA in GC cells. Furthermore, we observed a negative correlation between the expression levels of Wnt/β-catenin related stemness markers, such as CD44 and SOX9, and those of RPRD1A. The Wnt/β-catenin pathway is widely acknowledged as a growth regulatory pathway that plays crucial roles in various biological processes, including embryonic development, stem cell maintenance, cellular renewal, and adult tissue homeostasis. Numerous T cell factor 4 (TCF4)-binding sites are present in the Wnt/β-catenin signaling pathway. Nuclear β-catenin and TCF4 synergistically promote the upregulation of cancer stem cell-like properties^42,43^. After administering LF3, we conducted CO-IP and other experiments, and the results confirmed that RPRD1A impedes the formation of TCF4-β–catenin transcription complex. Therefore, it is hypothesized that RPRD1A may exert its downregulatory effect on molecules such as SOX9 via the Wnt/β-catenin signaling pathway, thereby inhibiting the stem cell-like properties of GC and enhancing sensitivity to chemotherapeutic agents. We provided the potential strategy for patients with *Circ-0075305*/*miR-708-5p*/RPRD1A pathway inhibition via WNT signaling pathway inhibitors.

In GC tissues, patients with high *Circ-0075305* and RPRD1A expression showed significantly improved OS rates after postoperative chemotherapy. Additionally, we established stable GC cell lines, tumor spheres, and organoids expressing or knocking down *Circ-0075305* and RPRD1A to fully validate the aforementioned hypothesis. Although OXA has demonstrated efficacy in treating advanced GC, the development of chemoresistance often results in poor outcomes. Therefore, it is imperative to investigate the key molecules involved in chemotherapy resistance and analyze their specific mechanisms.

Our study demonstrated that *Circ-0075305* can suppress the stem cell-like properties of GC cells. Meanwhile, the correlation among *Circ-0075305*, *miR-708-5p*, RPRD1A and Wnt/β-catenin related stemness markers (SOX9, SOX2, and CD44) was also observed in GC cells. Furthermore, *Circ-0075305* was demonstrated to enhance the sensitivity of GC to OXA chemotherapy by up-regulating the expression of RPRD1A as a target gene of *miR-708-5p* and inhibiting the transcriptional activity of stem cell markers such as SOX9 via the Wnt/β-catenin signaling pathway.

During the preliminary experiments in the early stages of our research, we found that among various gastric cancer cell lines, BGC-823 has the highest level of *Circ-0075305* mRNA. This realization holds important research implications, hence we chose BGC-823 as one of the cell lines for our study. To comprehensively validate our conclusions, in the subsequent phases of our study, an exhaustive experimental validation was performed on other cell lines including HGC-27, and gastric cancer organoids, murine orthotopic carcinoma models, as well as human gastric carcinoma tissue specimens. Our deployment of the contentious BGC-823 as one of our research subjects has resulted in our arguments being less robust than we would prefer. We anticipate conducting further studies on various cell lines to bolster experimental evidence. In spite of the fact that our laboratory is proficient in the induction of in situ carcinogenesis in mice as well as the creation of genetically engineered mice, we’ve encountered setbacks in the fabrication of genetically engineered mice owing to the peculiarities of CircRNA. This has, regrettably, curtailed our opportunities for a further exploration into the operational mechanism of *Circ-0075305* within the genetically-engineered mice. In this regard, our ensuing work will accentuate investigations pertaining to CircRNA genetically engineered mice. A holistic understanding of the role and modus operandi of Circ-0073505 in the etiology and advance of GC will undoubtedly enhance our comprehension of the biology of GC stem cell-like properties. It could potentially also catalyze further advancements in treatments for OXA chemoresistant cases. Furthermore, our work opens up fresh avenues for therapeutic interventions in cancer treatment by identifying innovative, suitable therapeutic targets, which could produce a important theoretical contribution to the regulatory networks governing cancer stem cell-like characteristics.

## Methods

### CircRNA microarray analysis

CircRNA microarray assay analyzed gastric cancer cell HGC-27 and gastric normal Gastric epithelial cell GES-1, as well as HGC-27 with OXA resistance. CircRNA microarray detection, collection, and analysis were performed by Nuohe Zhiyuan Technology, Beijing, China. CircRNAs were considered differentially regulated if they exhibited a fold change of ≥1.5 and a *P*-value < 0.05. We preliminarily screened 208 downregulated CircRNAs and 45 downregulated CircRNAs, and conducted cross analysis.

### Human tissue samples

A total of 163 pairs of matched gastric tumor tissues were collected from Fujian Medical University Union Hospital between 2014 and 2016, and all samples underwent qRT-PCR analysis. The study adhered to the principles outlined in the Declaration of Helsinki, with inclusion criteria consisting of pathological diagnosis as GC, complete clinicopathological features and five-year follow-up information, and TNM staging strictly following the 2010 UlCC guidelines. Informed consent agreements were obtained from all individual patients, and this work was reviewed and approved by the ethics committee of Fujian Medical University Union Hospital (No. 2020KY016). All ethical regulations relevant to human research participants were followed.

### RNA isolation and qRT-PCR

Total RNA was extracted using TRIzol reagents (Invitrogen, Carlsbad, CA, USA) according to the standard RNA extraction protocol. Reverse transcription was performed with the ReverTra Ace qPCR RT kit (TaKaRa, Dalian, China) to obtain cDNA. The Thunderbird SYBR qPCR mixture (TaKaRa), recommended by the manufacturer, was utilized for RT-qPCR analysis on the ABI Prism 7500 sequence detection system (Thermo Fisher Scientific). The primer sequences are provided below: *Circ-0075305* (5′ATTACGGGCTCAACATGCCA 3′, 5′GCATCACATGGGCAGTAGGA 3′). *miR-708-5p* (5′caCAGCUAGGGUCUUCCUAGCAGCUCCUc 3′, 5′ggGUCGAUCU---AA-CAU--UCGAGGAa 3′). GAPDH (5′GAAGGTGAAGGTCGGAGT 3′, 5′GAAGATGGTGATGGGATTTC 3′). Primer was provided by Biosune (Shanghai, China).

### Fluorescence in situ hybridization (FISH)

*Circ-0075305*, *miR-708-5p*, and RPRD1A probes were designed by Wuhan Bioxavier. The probes were incubated in a constant temperature water bath at 75°C for 5 minutes and immediately denatured at 0 °C for 5–10 min. After the specimens were roasted, denatured, dehydrated, and dried, 10 µL of denatured or pre-annealed DNA probe was applied to the denatured and dehydrated slide specimens for hybridization. The DNA probe was then sealed and allowed to hybridize overnight at 37°C (approximately 15–17 h) in a damp cassette. Following elution with formamide/SSC, 200 µL of redyeing solution (PI/antifade or DAPI/antifade) was added. After signal amplification, the plates were sealed and the expression levels and distributions of *Circ-0075305*, *miR-708-5p* and RPRD1A were observed using a laser confocal microscope Leica SP5 (Leica).

### Cell proliferation and invasion assays

A total of 2000 transfected cells were seeded into each well of a 96-well plate (Biosciences, USA) and incubated for 0, 24, 48, 72, and 96 hours. At different time points, CCK-8 reagent (10 µl) was added to each well and incubated at 37 °C for one hour. Transwell systems and Matrigel from BD Biosciences (New York, NY, USA) were utilized as per the manufacturer’s instructions. After a two-day incubation period, the cells were stained with crystal violet solution (0.5%) followed by microscopic counting using Olympus Corporation equipment.

### Tumor spheroid culture

Cells were seeded into ultra-low attachment 6-well dishes (Corning Life Sciences, NY, USA) and cultured in serum-free DMEM-F12 containing 20 ng/ml epidermal growth factor (EGF), 10 ng/ml basic fibroblast growth factor (bFGF), 2% B-27 (Life Technologies, Gaithersburg, MD, USA), and 2 mM L-glutamine (Life Technologies, Gaithersburg, MD, USA) as previously described. Spheroids were incubated in a 5% CO_2_ chamber at 37 °C for seven days. The culture medium was changed every three days. The diameter and number of tumor spheres in three random magnification fields were calculated under All-in-one Fluorescence Microscope (BZ-X700, Keyence Corp, Atlanta, GA, USA) in the bright light model. Spheroids were collected after 7 days except when noted otherwise. Protein was extracted for analysis, or cells were dissociated with Accutase (Innovative Cell Technologies, San Diego, CA, USA) and used for other experiments.

### Western blot analysis

RIPA buffer (middle) (Shanghai Beyotime, China) is used to lyse tissues and cells. 40 μg of protein was loaded into each pore, separated by 10% SDS-PAGE, and subsequently transferred onto a polyvinylidene fluoride membrane (EMD Millipore, Billerica, MA, USA). Following blocking, the membrane was incubated with primary antibody overnight in a dilution buffer specifically designed for primary antibodies (Thermo Fisher Scientific, Shanghai, China). Following TBST washing, the membrane was subjected to incubation with corresponding antibodies at room temperature for 1 hour and subsequently washed thrice with TBST. The proteins on the membranes were visualized using enhanced chemiluminescence (Amersham; GE Healthcare). Experiments were performed in triplicate. Utilize the ImageJ software for the analysis of grayscale values in each strip. Each experimental group is compared to the control group as a reference, and the relative multiplication relationship is subsequently computed. Uncropped blots are shown in Supplementary Fig. 13.

### Sample collection, fixation and sectioning

Paraffin sections were dewaxed, washed in distilled water and soaked in PBS for 5 min. 3% O2 at room temperature 2 H incubation for 10 min, eliminate endogenous peroxidase activity; Wash in distilled water and soak in PBS for 5 min. Appropriately diluted antibodies were added to Ki 67 (1:2000, abcam), RPRD1A (1:1000, Proteintech), CD44 (1:1000, abcam), and SOX9 (1:1000, abcam) and incubated at 37 °C for 1–2 h or 4 °C overnight. Rinse with PBS, 3 × 3 min. Add polymer Helper and incubate at room temperature or 37 °C for 20 min. Wash with PBS, 3 × 3 min. Drop in polyperoxidase- anti-rat/rabbit IgG (the second antibody) polymer and incubate at 37 °C or room temperature for 20–30 min. Rinse with PBS, 3 × 3 min. DAB color development, control color development degree under microscope, generally 3–10 min. Fully washed with distilled water, restrained with hematoxylin and sealed.

### Actinomycin D assay

HGC-27 and BGC-823 were cultured in 2 µg/mL actinomycin D (Sigma) to inhibit transcription for 4, 8, 12, 16, 20, and 24 h. The cells were harvested, and the stability of *Circ-0075305* and MAML1 mRNA was assessed by qRT-PCR analysis.

### RNase R digestion

Total RNA (2 µg) was incubated at 37 °C for 20 min, and two sets were designed with or without 3 U/µg RNase R The RNA obtained was purified through the utilization of RNeasy MinElute cleansing kit (Qiagen) and subsequently detected by qRT-PCR.

### Nucleocytoplasmic separation

A gastric cancer cell line (HGC-27) overexpressing/knocking down RPRD1A was extracted using Nuclear and Cytoplasmic Protein Extraction Kit (biosystems P0028). western blot was used to detect whether the expression of target proteins β-catenin, TCF4 and RPRD1A in the nucleus and cytoplasm was affected by RPRD1A expression level.

### Flow cytometry assay

The stably transfected gastric cancer cells were digested and centrifuged and placed in 1.5 ml tubes. The cells were washed three times with PBS and centrifuged at 1000prm for 5 min. This supernatant was discarded and subsequently added to 100 μl staining buffer (PBS, pH 7.4, 0.1% BSA) containing 1 μg/ml CD44 antibody (Abcam) and incubated at 4 °C for 30 min. The cells were subsequently resuspended in PBS without undergoing washing and subjected to collection on a FACS flow cytometer as per the manufacturer’s instructions. The results obtained were analyzed using FlowJo software. We used a multi-gating strategy to identify and distinguish different cell populations by setting the same standard FSC (forward scattered light) and SSC (side scattered light), determine the target cell population for our study, and finally detect the cell proportion of CD44 + . The flow cytometry gating strategy is shown in Supplementary Fig. 14.

### Oxaliplatin-resistant gastric cancer cell lines

Oxaliplatin-resistant HGC-27 (HGC-OXY) cells were established by the Laboratory of Gastrointestinal Cancer, Fujian Medical University. HGC-27 cells were treated with OXA by a stepwise increase in drug concentration from 1 to 100 μM (1, 20, 40, 60, 80, 100) every one week, until HGC-27 cells became resistant to 100 μM of OXA.

### Organoid culture

Human organoids culture was performed following previously published protocol. Briefly, tumor tissues from the stomach were washed with PBS containing 1× Penicillin/Streptomycin twice (BL505A, Biosharp, Hefei, China), followed by removing the muscle layer and mucus using scissors. Subsequently, the sample should be sliced into 2–3 mm sections and subjected to enzymatic digestion using 2.5 mg/ml of Collagenase A. (Sigma-Aldrich, St. Louis, MO, USA) for 30 mis. 5 ml Dissociation buffer, containing D-sorbitol (Sigma-Aldrich, St. Louis, MO, USA) and sucrose (Sigma-Aldrich, St. Louis, MO, USA), was added to the tissue and agitated for 2 minutes. The final supernatant was filtered through a 70μm mesh and the crypts fraction was centrifuged at 150 g for 5 min. After being washed with ice-cold PBS, the gland pellet was resuspended in Matrigel™ (356255, Corning, USA) supplemented with standard gastric organoid advanced DMEM/F12 (#12634010, Thermo Fisher Scientific, Waltham, MA, USA), 1× GlutaMax (#35050061, Thermo Fisher Scientific, Waltham, MA, USA), 1× HEPES (#15630080, Thermo Fisher Scientific, Waltham, MA, USA), 1× Penicillin/Streptomycin, 50% Wnt3a, 10% RSPO-1, 10% Noggin, 1× B27 (#17504001, Thermo Fisher Scientific, Waltham, MA, USA), 50 ng/mL EGF (PHG0311, Thermo Fisher Scientific, Waltham, MA, USA), 200 ng/mL FGF10 (#100-26, Peprotech, Rocky Hill, NJ, USA), 1mM N-acetyl-L-cysteine (#A9165, Sigma-Aldrich, St. Louis, MO, USA), 1 nM Gastrin (#G9145, Sigma-Aldrich, St. Louis, MO, USA), 2 mM A83-01 (#2939/10, Tocris, Bristol, UK), 10 mM Y-27632 (#1254/10, Tocris Bristol, UK). Finally, 50 μl Matrigel™ suspension was carefully ejected into the center of each well of the 24-well plate. 1 ml of standard gastric organoid medium was added to each well. The organoids were cultured in a 5% CO2 incubator at 37 °C and the media was changed every 2–3 days. Organoids from the second passage were infected with lentivirus carrying either control or RPRD1A overexpression in 15 ml tubes overnight. Seven days after infection, the diameter and number of organoids were measured under a light microscope in three random fields magnified at 100×. For histological examination of the organoids, they were fixed in 4% paraformaldehyde for 1 hour and subsequently embedded in a 2% agarose gel or directly fixed in formalin-containing Matrigel to generate paraffin blocks for sectioning and staining.

### Tumor xenograft assay

BALB/c nude mice were randomized and performed under standard conditions in accordance with the established guidelines of Fujian Medical University Animal Laboratory Center. The Institutional Animal Care and Use Committee of Fujian Medical University Union Hospital approved the animal experiments. We have complied with all relevant ethical regulations for animal use. All BALB/c nude mice (4-6 weeks of age) used in our study were purchased from Beijing Vital River Laboratory Animal Technology Co., Ltd. To evaluate the impact of *Circ-0075305* on stemness, limiting dilution assays were performed in nude mice.Cells were injected subcutaneously into the right axillary fossa of nude mice at indicated cell concentrations. Five mice were used in each experimental group. Tumor formation was checked every 3–4 days and the mice were sacrificed at 4–6 weeks after injection and the tumors were weighed and used in immunohistochemical staining studies. Tumor volume was calculated with the following formula: V = (L × W2)/2 (V, tumor volume; L, length; W, width), and growth curves were plotted using average tumor volume within each experimental group at the set time points. The frequency of tumor-initiating cells was calculated using the extreme limiting dilution analysis program (http://bioinf.wehi.edu.au/software/elda/)^44^.

### Immunohistochemistry (IHC)

According to PV-9000 General Two-step Detection Kit (Zhong Shan-Golden Bridge Biological Technology Co., Ltd., Beijing, China) instructions constructed IHC staining. Sections were then counterstained with hematoxylin, dehydrated, and mounted. Staining intensities and extents of CD44 and SOX9 expression were graded as follows: negative (score 0), weak (score 1), moderate (score 2), and strong (score 3). Percentage scores were assigned as 1–25, 1–25%; 26–50, 26–50%; 51–75, 51–75%; and 76–100, 76–100%. The scores of each tumor sample were multiplied to give a final score of 0–300. Immunohistochemical scores were evaluated by two independent pathologists, and in case of disagreement, a third pathologist would be invited to discuss and reach a unanimous decision.

### Statistics and reproducibility

The overarching sample sizes of randomly collected clinical information of gastric cancer patients in Fujian Medical University Union Hospital from 2014 to 2016 was 200. The statistical analysis was done using SPSS 25.0 software (IBM, Chicago, IL). Results are presented as mean ± SD and differences were assessed by Student’s t-test or one-way analysis of variance (ANOVA) as appropriate. Survival rates were calculated with the Kaplan-Meier method and analyzed by log-rank test. The shaded areas indicate the 95% confidence intervals. The results were presented as means and the error bars represented the standard deviation. *P < 0.05, **P < 0.01, ***P < 0.001 for groups connected by horizontal lines. *P*-values < 0.05 were considered statistically significant. Error bars show standard error of the mean. Bar graphs show mean ± SD and individual data points. The source data of the article are deposited in Supplementary Data 1.

### Ethical consideration

This study was approved by the institutional review board of each participating center. Informed consent agreements were obtained from all individual patients, and this work was reviewed and approved by the ethics committee of Fujian Medical University Union Hospital (No. 2020KY016). The Institutional Review Committee approved all the experimental protocols using de-identified All animal experiments were performed in accordance with the protocols approved by the Animal Experimentation Ethics Committee of Fujian Medical University (IACUC FJMU 2020-0299).

### Reporting summary

Further information on research design is available in the Nature Portfolio Reporting Summary linked to this article.

### Supplementary information

Supplementary Information

Description of Additional Supplementary Files

Supplementary Data 1

Reporting Summary

## Data Availability

The data supporting the findings in this study are available under controlled access due to data privacy laws related to patient consent for data sharing and the data should be used for research purposes only. All the original clinical data will be made available on request from the corresponding author (Huang CM) at any time in a de-identified manner. All public and sequencing data for this study have been uploaded to Figshare (10.6084/m9.figshare.25351414). The remaining data are available within the Article, Supplementary Information. Request for data sharing will be handled in line with the data access and sharing policy of Fujian Medical University Union Hospital. The source data can be found in Supplementary Data 1.
