# Supplementary material for: Circ-0075305 hinders gastric cancer stem cells by indirectly disrupting TCF4–β-catenin complex and downregulation of SOX9
Source: Commun Biol. 2024 May 7;7:545. doi: 10.1038/s42003-024-06213-6 (PMC11076483; doi:10.1038/s42003-024-06213-6)

Supplementary Information

Article Title

Circ-0075305 hinders gastric cancer stem cells by indirectly disrupting TCF4–β-catenin complex and downregulation of SOX9

Authors

Qi-Yue Chen[1,2,3#], Kai-Xiang Xu[1,2,3#], Xiao-Bo Huang[1,2,3#], Deng-Hui Fan[1,2,3#], Yu-Jing Chen[1,2,3], Yi-Fan Li[1,2,3], Qiang Huang[1,2,3], Zhi-Yu Liu[1,2,3], Hua-Long Zheng[1,2,3], Ze-Ning Huang[1,2,3], Ze-Hong Lin[1,2,3],Yu-Xiang Wang[1,2,3], Jun-Jie Yang[1,2,3], Qing Zhong[1,2,3*], Chang-Ming Huang[1,2,3*]

Authors' affiliations

1. Department of Gastric Surgery, Fujian Medical University Union Hospital, Fuzhou, China.2. Department of General Surgery, Fujian Medical University Union Hospital, Fuzhou, China.3. Key Laboratory of Ministry of Education of Gastrointestinal Cancer, Fujian Medical University, Fuzhou, China.

Corresponding author

Chang-Ming Huang, E-mail: hcmlr2002@163.com; Qing Zhong, E-mail: 845733977@qq.com; Department of Gastric Surgery, Fujian Medical University Union Hospital, No. 29 Xinquan Road, Fuzhou 350001, Fujian Province, China. Telephone: +86-591-83363366, Fax: +86-591-83363366

Supplementary Figure Legends

Supplementary Figure 1. Screening of Circ-0075305 and unique properties of CircRNA.
(a) Flowchart showing screening of candidate CircRNAs orchestrating GC stemness. The overlap of down-regulated CircRNAs between gastric cancer cells and normal gastric epithelial cells, as well as between gastric cancer cells and chemoresistant gastric cancer cells. (b) Stability of CircRNA in BGC-823 cells was verified using an actinomycin D assay. (c) The characteristics of CircRNAs that were not easily digested by BGC-823 were verified using RNase R exonuclease digestion experiments. (d) The locations of CircRNAs were verified by performing nucleoplasmic separation experiments in BGC-823. (e) The cyclic properties of CircRNAs in BGC-823 cells were verified by DNA gel electrophoresis. (f) The location of Circ-0075305 (green) in the cytoplasm of GC cells was verified by FISH. Cell nucleuses were counterstained with DAPI (blue). Data are represented as the mean ± SD and analyzed by Student's t-test. NS, no significance, *P <0.05, **P <0.01, *** P <0.001 for groups connected by horizontal lines. P-values < 0.05 were considered statistically significant. b, c, d: n = 3 per group.

Supplementary Figure 2. Effect of Circ-0075305 on proliferation and migration of GC in vitro.
(a) Representative image of colony formation assay in BGC-823 and HGC-27 cells. (b) Statistical analysis of the results of cell colony formation assays in Supplementary Fig. a. (c) Representative images of cell migration assay in BGC-823 and HGC-27 cells. (d) Statistical analysis of the results of migration assays in Supplementary Fig. c. (e) Representative images of invasion assay in BGC-823 and HGC-27 cells. (f) Statistical analysis of the results of invasion assays in Supplementary Fig. e. Data are represented as the mean ± SD and analyzed by Student's t-test. **P <0.01, *** P <0.001 for groups connected by horizontal lines. P-values < 0.05 were considered statistically significant. b, d, f: n = 3 per group.

Supplementary Figure 3. Effect of Circ-0075305 on proliferation and migration of GC in vivo.
(a-b) Tumor formation by Circ-0075305 GC cells inoculated subcutaneously into mice. (c) The changes of tumor volume were recorded. (d) The changes of tumor weight were recorded. (e) The expression and distribution of HE and Ki-67 in these tumors were detected using immunohistochemistry. (f) Statistical analysis of Ki-67 positive cells in tumor tissues. (g) A metastasis model was constructed using overexpressed Circ-0075305 GC cell lungs, and metastasis was analyzed by in vivo imaging and HE staining. (h) A metastasis model was constructed using overexpressed Circ-0075305 GC cells, and metastasis was analyzed by in vivo imaging and HE. Data are represented as the mean ± SD and analyzed by Student's t-test. **P <0.01, *** P <0.001 for groups connected by horizontal lines. P-values < 0.05 were considered statistically significant. f: n = 3 per group; c, d: n = 5 per group.

Supplementary Figure 4. In Vitro study of the effect of up-regulation of Circ-0075305 on the reduction of chemotherapy sensitivity of GC cells.
(a) Schematic diagram of human GC tumor spheres and organoids treated with OXA. (b) Survival of HGC-27 tumor spheres treated with OXA after stable rotational expression and silencing of Circ-0075305. (c) The proportion of GC ball survivors (Supplementary Fig. b was statistically analyzed). (d) Survival of BGC-823 tumor spheres treated with OXA after stable transmutation and

silencing of Circ-0075305. (e) Statistical analysis of the proportion of GC ball survival in Supplementary Fig. d. (f) Organoid survival of Circ-0075305 GC cells after OXA treatment, with stable transversal expression and silencing. (g-h) The number and proportion of surviving GC organoids Supplementary Fig. f were statistically analyzed. Data are represented as the mean ± SD and analyzed by Student's t-test. *** P <0.001 for groups connected by horizontal lines. P-values < 0.05 were considered statistically significant. c, e, g, h: n = 5 per group.

Supplementary Figure 5. Effect of Circ-0075305 on stem cell-like properties of GC in vitro. (a-b) Stably transfected Circ-0075305 overexpressed/knockdown Circ-0075305 tumor spheres were constructed, and the number changes of the above tumor spheres were detected and counted. (c-d) GC organoids stably transfected with overexpression or knockout of Circ-0075305 were constructed, and the size changes of these organoids were detected and counted. (e) Flow cytometry was used to assess the proportion of CD44+ cells in GC tumor spheres (HGC-27 and BGC-823) transfected with Circ-0075305 overexpression or knockdown. (f) Statistical analysis of the results of the flow cytometry analysis in Supplementary Fig. e. (g) Western blot was used to analyze the effect of Circ-0075305 on gastric cancer stem cell-like characteristics. (h) Quantitative interpretation of the data from Supplementary Fig. g. (i-j) Representative immunofluorescence images of CD44 and NANOG in GC tumor spheres (HGC-27 and BGC-823) transfected with a vector, Circ-0075305 overexpression or Circ-0075305 knockdown. (k-l) The proportions of CD44 and NANOG positive GC tumor spheres in Supplementary Fig. i and j were statistically analyzed. (m) The distribution of CD44 and SOX9 expression in tumors was detected by using immunohistochemistry. (n) Statistical analysis of CD44 and SOX9 positive cells in tumor tissues. Data are represented as the mean ± SD and analyzed by Student's t-test. *P <0.05, **P <0.01, *** P <0.001 for groups connected by horizontal lines. P-values < 0.05 were considered statistically significant. b, d, f, h, k, i, n: n = 3 per group; b: n = 5 per group.

Supplementary Figure 6. Screening the downstream target miR-708-5p of Circ-0075305. (a-b) The expression of the predicted binding miRNAs in overexpressed and knockdown Circ-0075305 gastric cancer cell lines was detected using qRT-PCR. (c) The box plot depicted the distributions miR-302b-3p expression in GC tissues and adjacent normal tissues. The lower and upper sides of the box are the lower and upper quartiles. The whiskers are the two lines outside the box, that go from the minimum to the lower quartile and then from the upper quartile to the maximum. Each dot indicated score of individual patients. (d) The box plot depicted the distributions miR-708-5p expression in GC and adjacent normal tissues from the TCGA database. The lower and upper sides of the box are the lower and upper quartiles. The whiskers are the two lines outside the box, that go from the minimum to the lower quartile and then from the upper quartile to the maximum. Each dot indicated score of individual patients. Data are represented as the mean ± SD and analyzed by Student's t-test. NS, no significance, *** P <0.001 for groups connected by horizontal lines. P-values < 0.05 were considered statistically significant.

Supplementary Figure 7. Screening for RPRD1A, the downstream target gene of miR-708-5p. (a-b) qRT-PCR was used to predict the expression of mRNA targets in BGC-823 and HGC-27 cells transfected with Circ-0075305 and the miR-708-5p mimic, either alone or in combination. Data are represented as the mean ± SD and analyzed by one-way analysis of variance (ANOVA). NS, no

significance, *P <0.05, **P <0.01 for groups connected by horizontal lines. P-values < 0.05 were considered statistically significant. a, b: n = 3 per group.

Supplementary Figure 8. Expression of RPRD1A in GC.

(a) The Gene Expression Omnibus (GEO) database was used to ascertain the expression of RPRD1A in human gastric and adjacent normal tissues. (b) The GEO database was used to detect the expression of RPRD1A in mouse gastric and adjacent normal tissues. (c) Western blot analysis was performed to determine the differential expression of RPRD1A in human gastric and adjacent normal tissues. (d) Quantitative interpretation of the data from Supplementary Fig. c. Data are represented as the mean ± SD and analyzed by Student's t-test. *P <0.05, **P <0.01, *** P <0.001 for groups connected by horizontal lines. P-values < 0.05 were considered statistically significant. a, b: n > 5 per group; d: n = 24 per group.

Supplementary Figure 9. Circ-0075305 regulates stem cell-like properties of GC by targeting miR-708-5p.

(a) A bioinformatics approach was employed to investigate the correlation between miR-708-5p expression in the TCGA database, tumorigenesis, and stem cell-like characteristics. (b-c) GSEA was used to analyze the correlation between the expression of miR-708-5p and OXA IC50 values in TCGA and ACRG databases. (d) GSEA analysis of the correlation between the expression of miR-708-5p and the Wnt signaling pathway. (e) GC tumor spheres were transfected with Circ-0075305 and/or miR-708-5p mimics, and their growth was observed. (f) GC organoids were transfected with Circ-0075305 and/or miR-708-5p mimics, and their growth was observed. (g) Statistical analysis of changes in the number of tumor spheres in Supplementary Fig. e. (h) Statistical analysis of organoid size changes in Supplementary Fig. f. (i-j) Immunofluorescence images of CD44 and NANOG were obtained from gastric cancer spheres transfected with a vector, Circ-0075305 overexpression, miR-708-5p mimic expression, or both Circ-0075305 overexpression and miR-708-5p mimic expression. (k-l) The proportions of CD44 and NANOG positive GC spheres in Supplementary Fig. i and j were statistically analyzed. Data are represented as the mean ± SD and analyzed by one-way analysis of variance (ANOVA). *P <0.05, **P <0.01, *** P <0.001 for groups connected by horizontal lines. P-values < 0.05 were considered statistically significant. g, h, k, i: n = 3 per group; b, c: n > 5 per group.

Supplementary Figure 10. Circ-0075305 regulates stem cell-like properties of GC by targeting RPRD1A.

(a-b) A bioinformatics approach was employed to investigate the correlation between RPRD1A and miR-708-5p expression in the TCGA database, tumorigenesis, and stem cell-like characteristics. (c) GSEA was used to analyze the correlation between the expression of RPRD1A and OXA IC50 values in ACRG databases. (d) GSEA analysis of the correlation between the expression of RPRD1A and the Wnt signaling pathway. (e-f) Western blot analysis was employed to detect the expression level of RPRD1A in GC cells transfected with a vector, Circ-0075305 overexpression, Circ-0075305 knockdown, miR-708-5p mimic and miR-708-5p inhibitor. (g) Statistical analysis Supplementary Fig. e. (h) Statistical analysis Supplementary Fig. f. (i) GC tumor spheres were transfected with Circ-0075305 and/or RPRD1A knockdown, and their growth was observed. (j) GC organoids were transfected with Circ-0075305 and/or RPRD1A knockdown, and their growth was observed. (k)

Statistical analysis of changes in the number of tumor spheres in Supplementary Fig. i. (l) Statistical analysis of organoid size changes in Supplementary Fig. j. (m-n) Immunofluorescence images of CD44 and NANOG were obtained from gastric cancer spheres transfected with a vector, Circ-0075305 overexpression, RPRD1A knockdown, or both Circ-0075305 overexpression and RPRD1A knockdown. (o-p) The proportions of CD44 and NANOG positive GC spheres in Supplementary Fig. m and n were statistically analyzed. Data are represented as the mean ± SD and analyzed by one-way analysis of variance (ANOVA). *P <0.05, **P <0.01, *** P <0.001 for groups connected by horizontal lines. P-values < 0.05 were considered statistically significant. g, h, k, i, o, p: n = 3 per group; c: n > 5 per group.

Supplementary Figure 11. Flow cytometry analysis of RPRD1A's regulation of GC stem cell-like properties.
(a) Flow cytometry was used to assess the proportion of CD44+ cells in HGC-27 and BGC-823 cells transfected with RPRD1A knockdown. (b) Statistical analysis of the results of the flow cytometry analysis in Supplementary Fig. a. Data are represented as the mean ± SD and analyzed by Student's t-test. **P <0.01 for groups connected by horizontal lines. P-values < 0.05 were considered statistically significant. b: n = 3 per group.

Supplementary Figure 12. Correlation of RPRD1A with CD44 and SOX9 in GC tissues.
(a) The expression and distribution of RPRD1A and CD44 in GC tissues of patients at our center were detected by using immunohistochemistry. (b) Immune scores were assessed for RPRD1A and CD44 and correlations were analyzed.

Supplementary Figure 13. Uncropped and unedited blot/gel images.

Supplementary Figure 14. The flow cytometry gating strategy.

Supplementary Figure 1

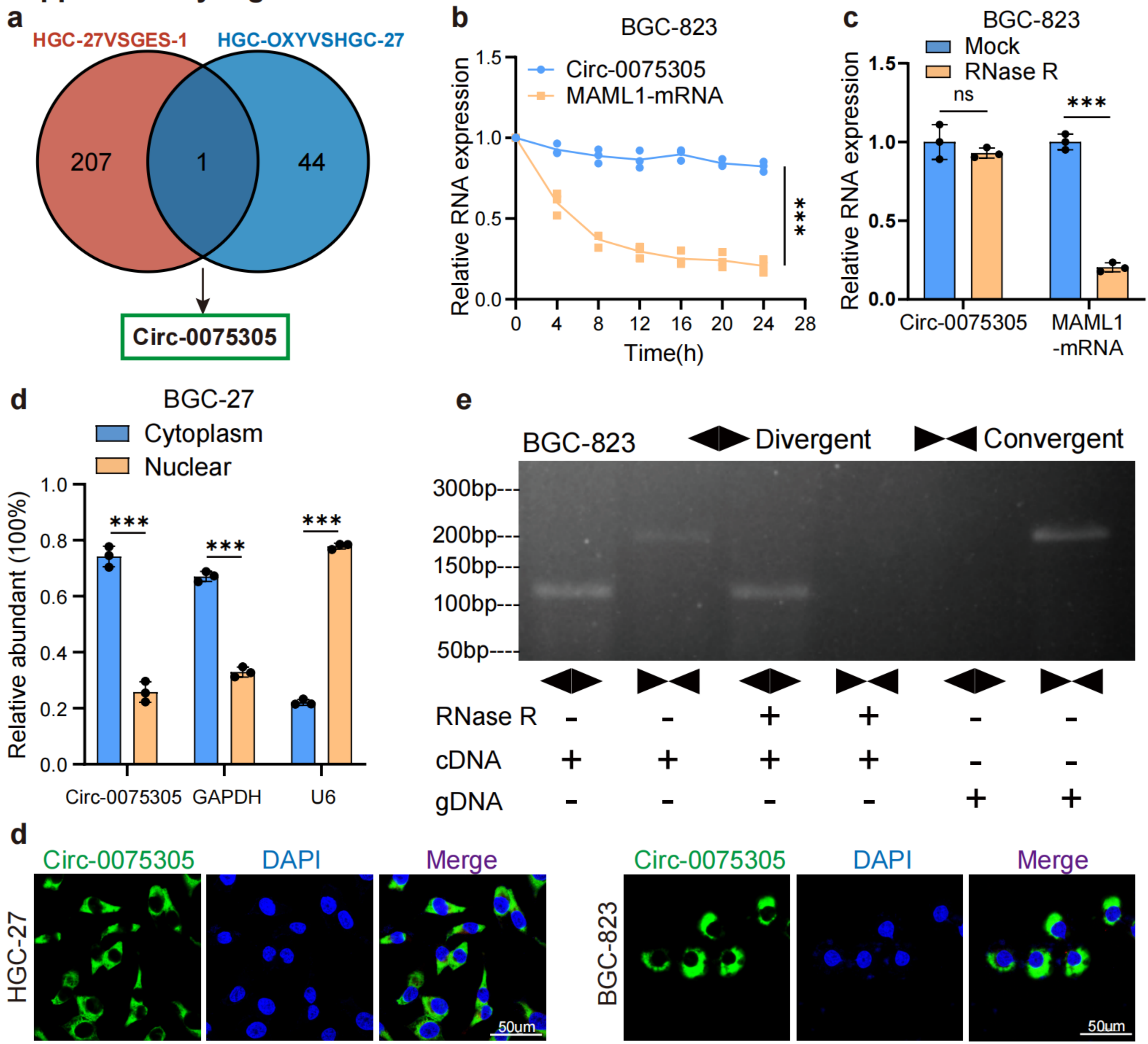

Supplementary Figure 2

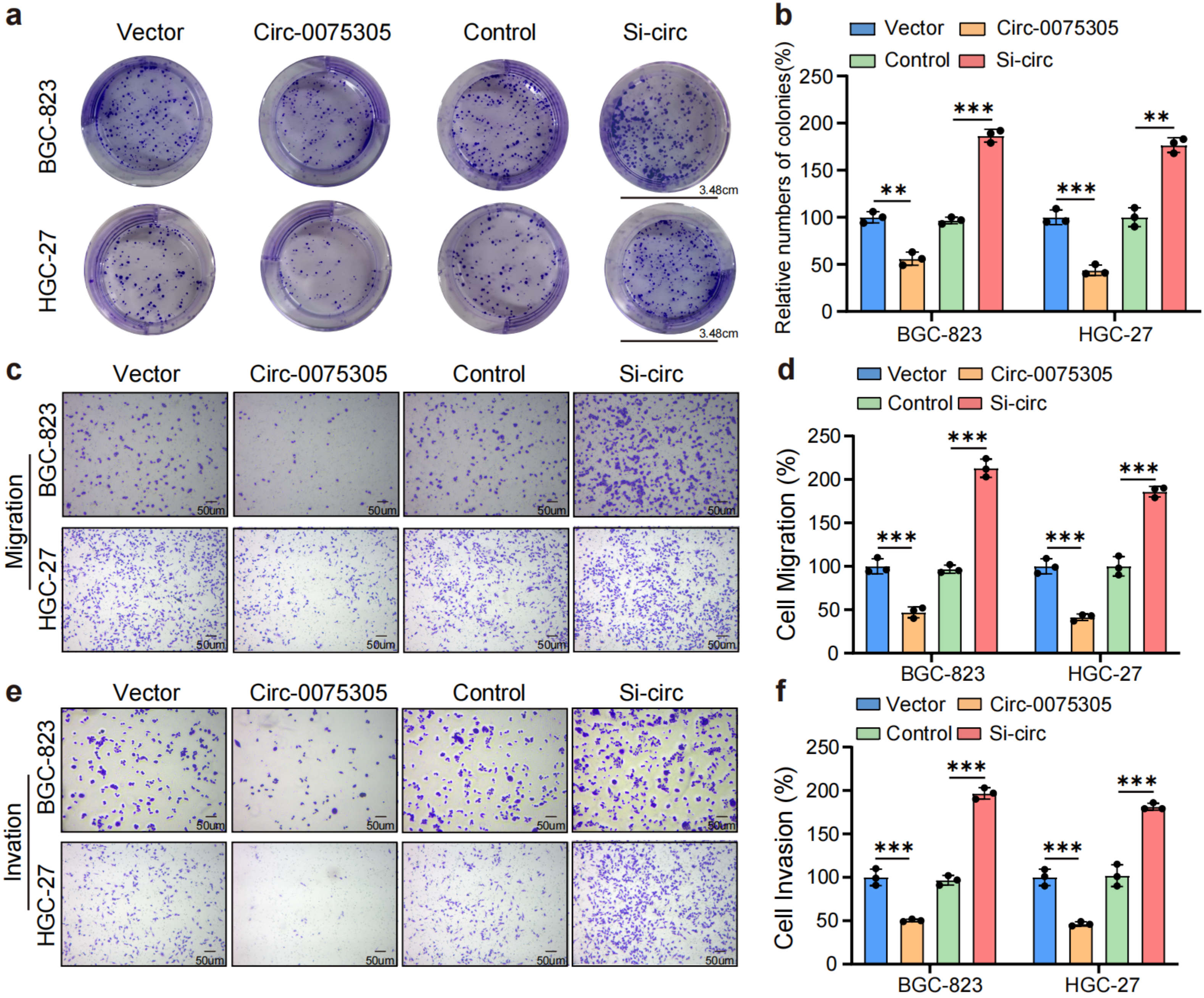

Supplementary Figure 3

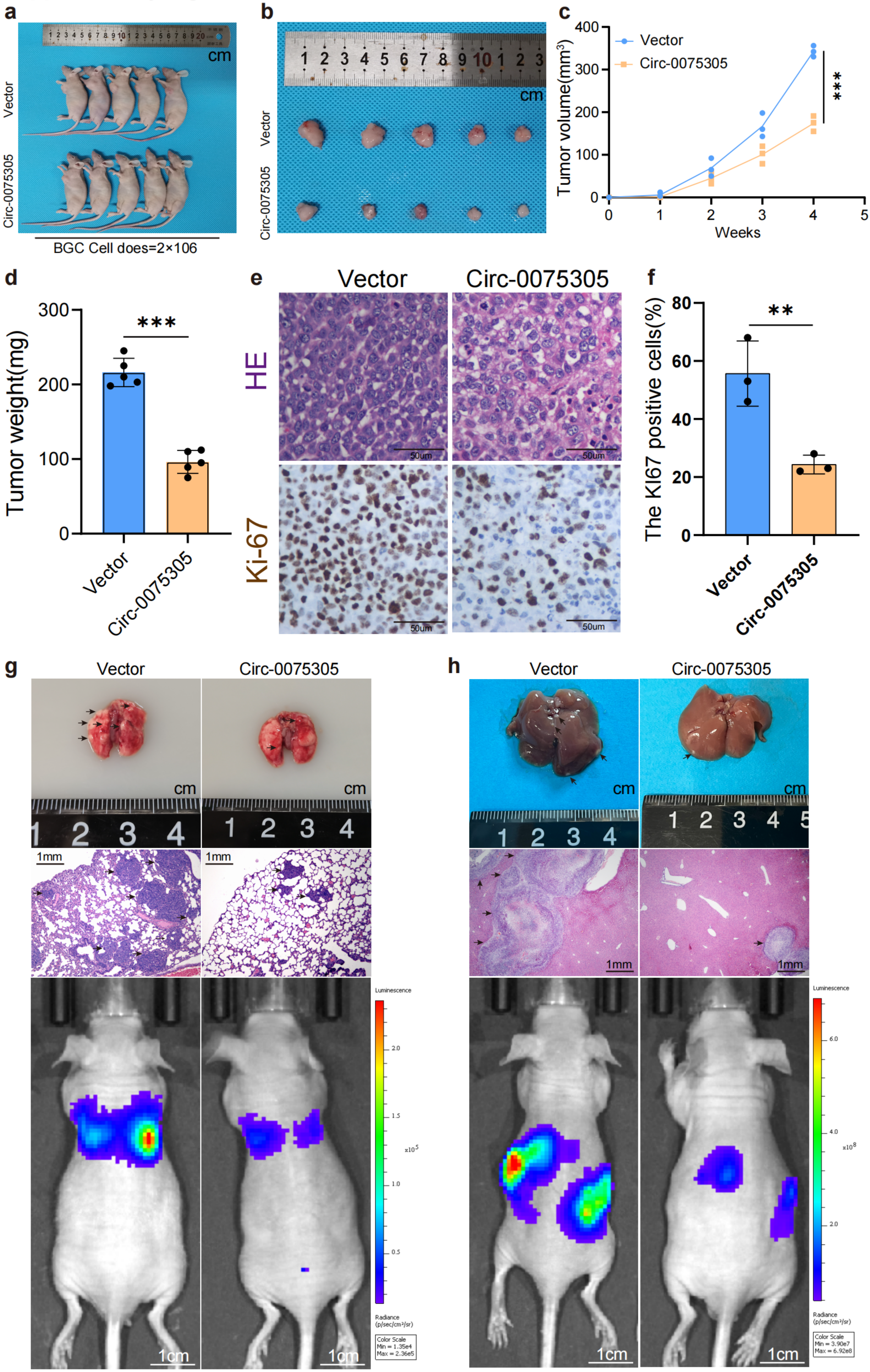

Supplementary Figure 4

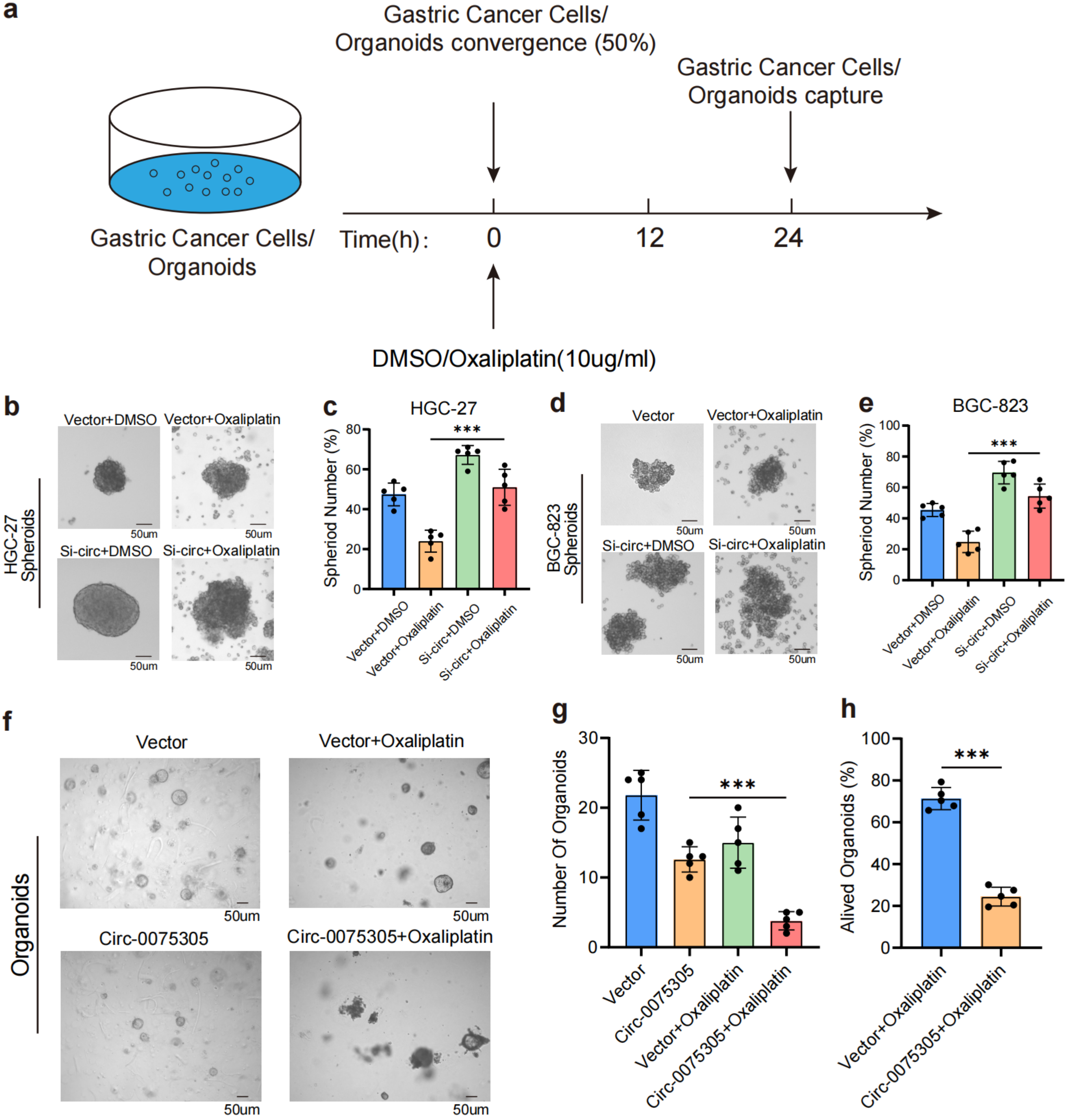

Supplementary Figure 5

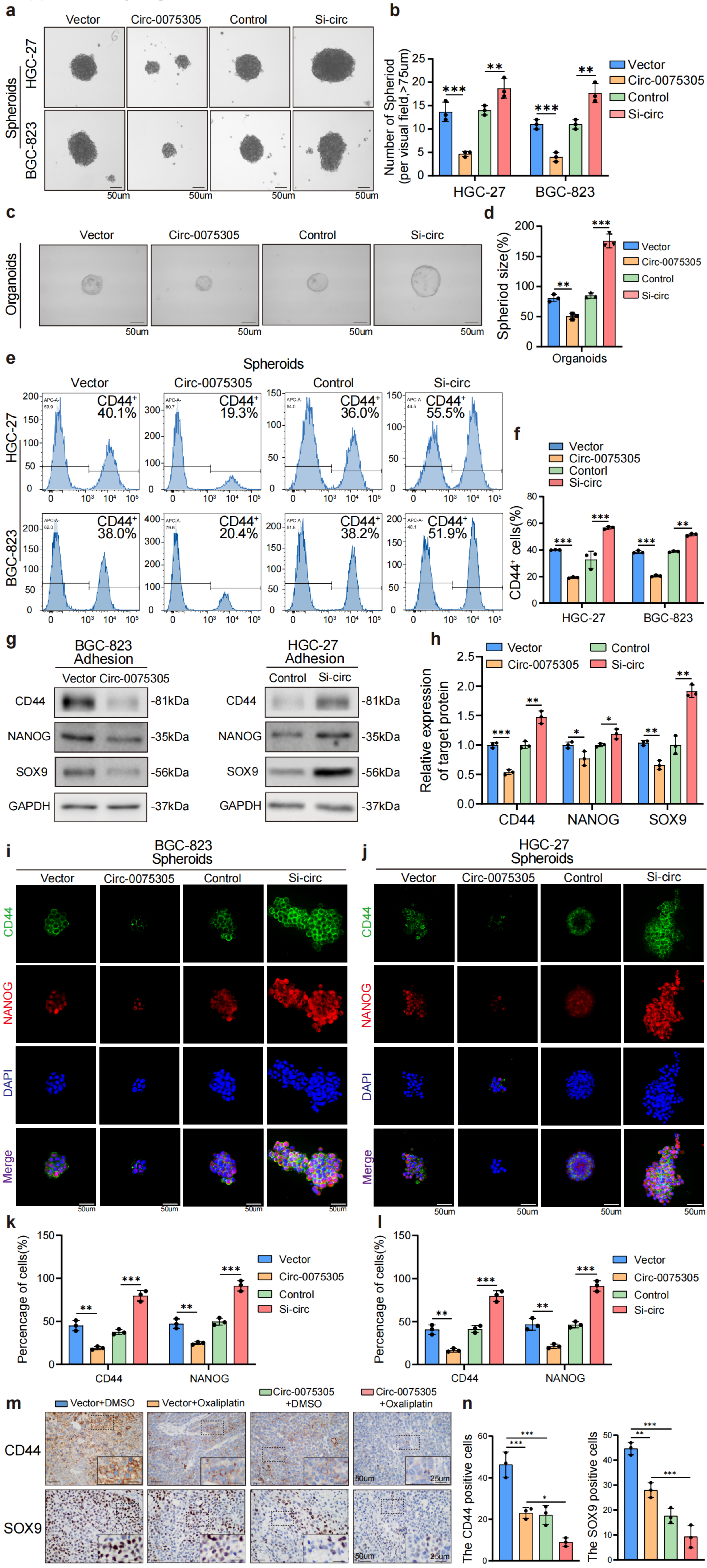

Supplementary Figure 6

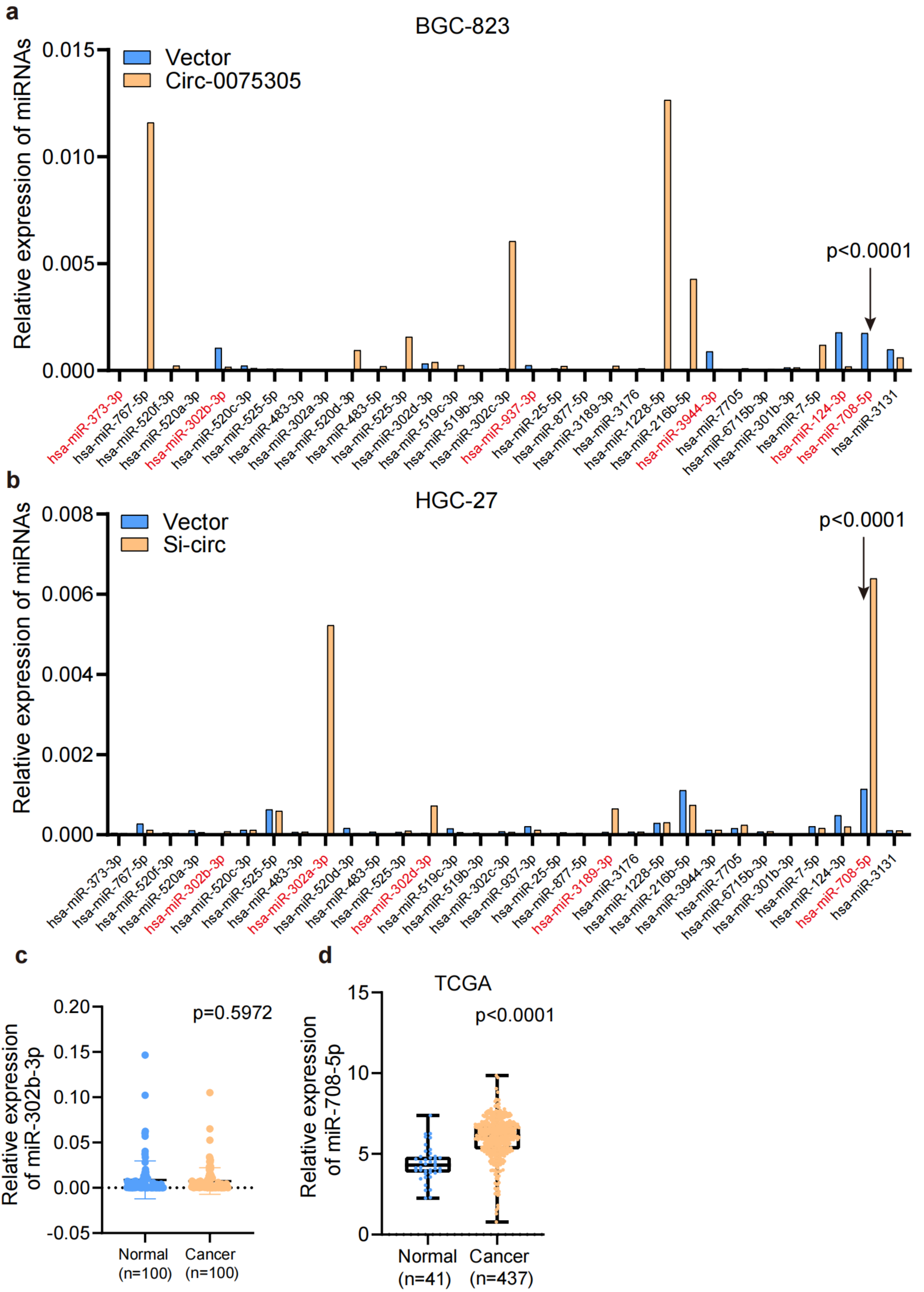

Supplementary Figure 7

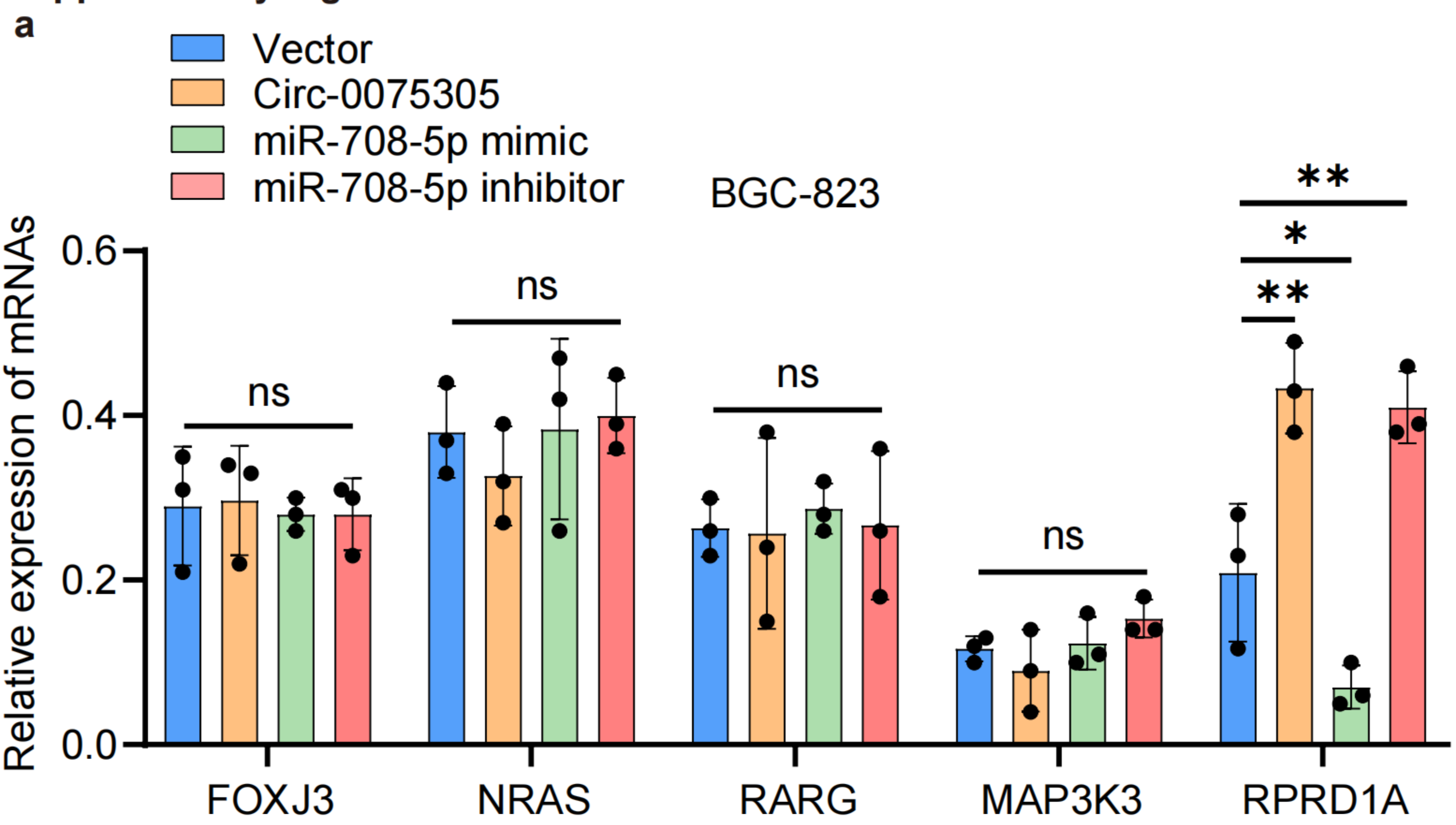

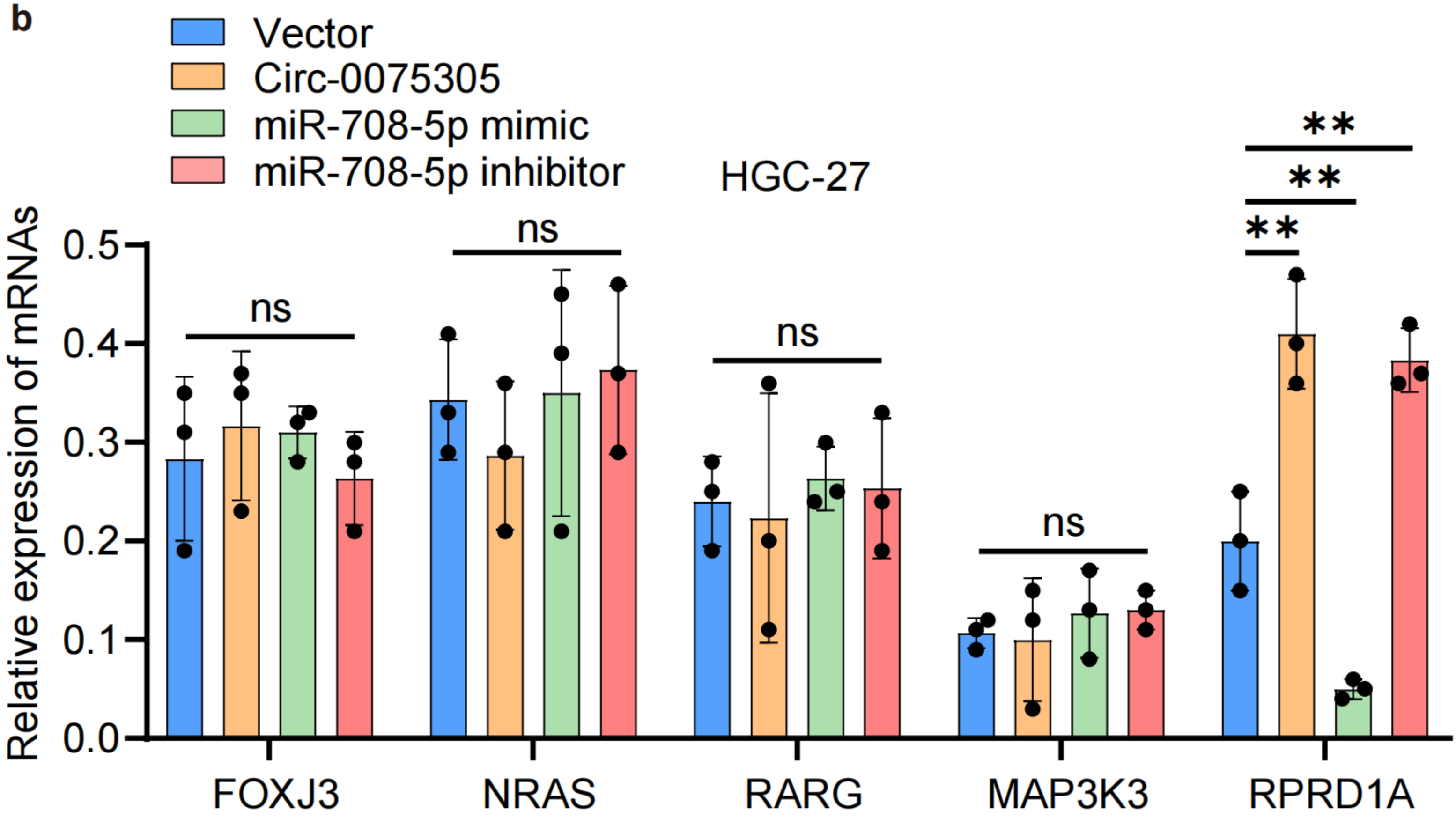

Supplementary Figure 8

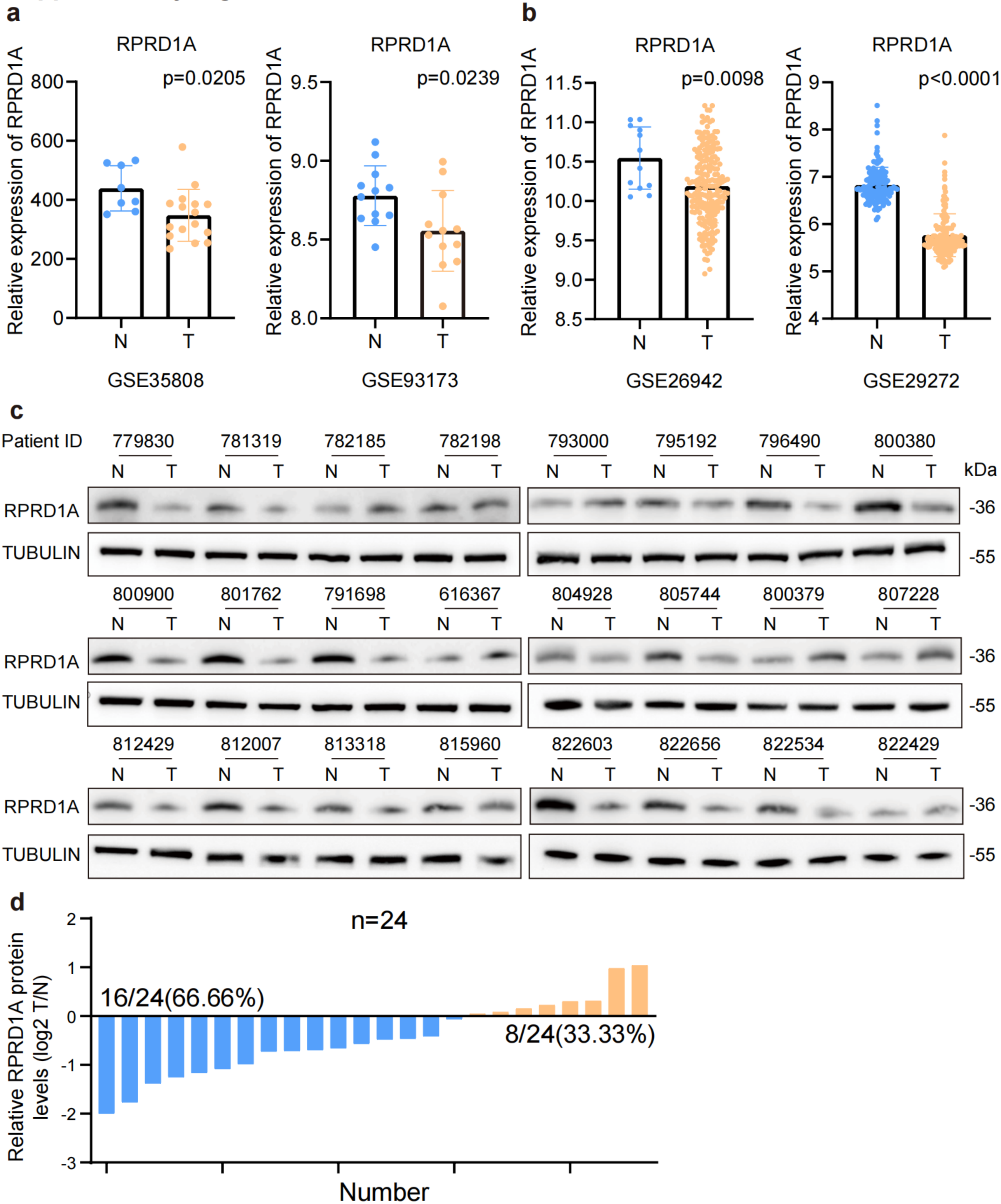

Supplementary Figure 9

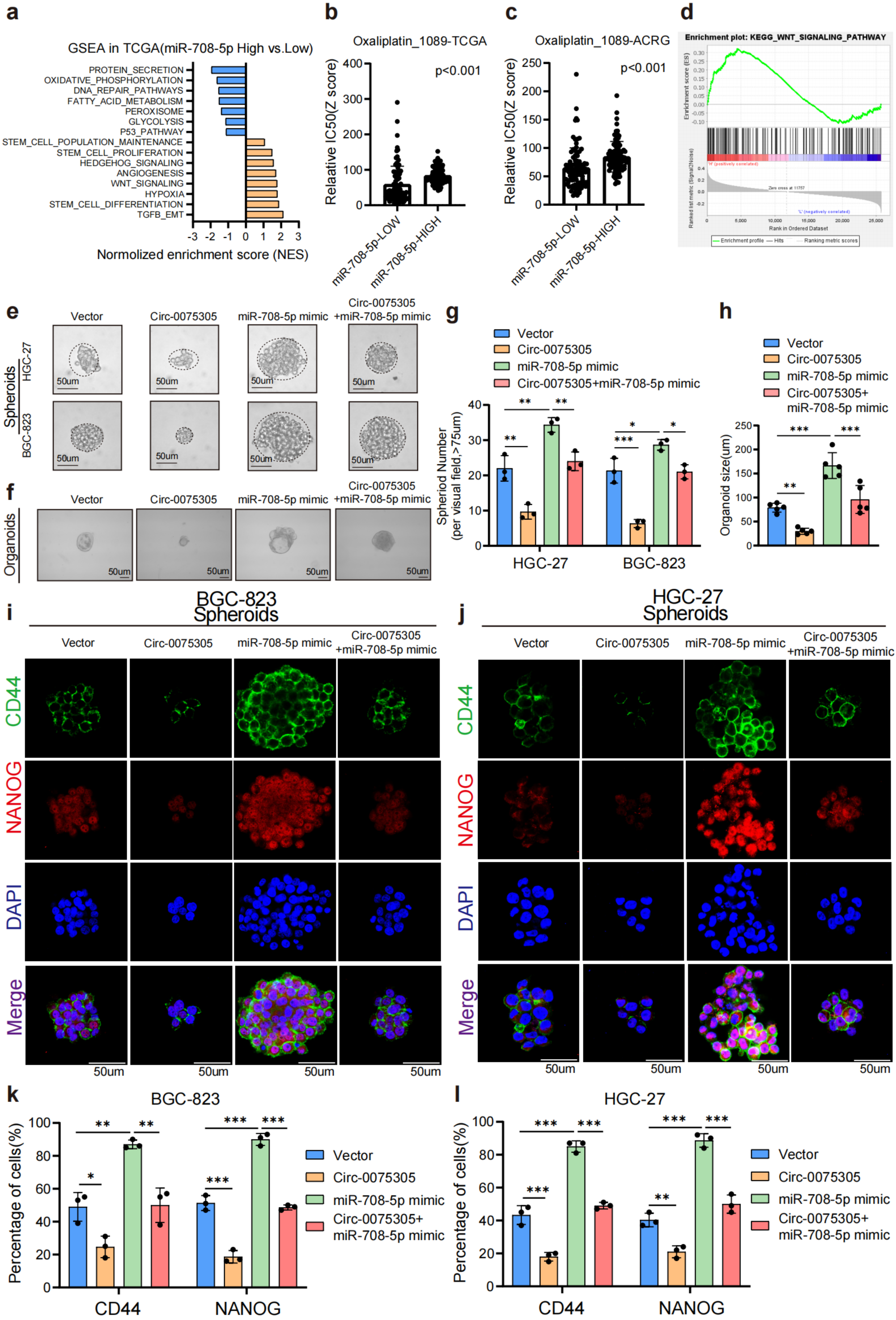

Supplementary Figure 10

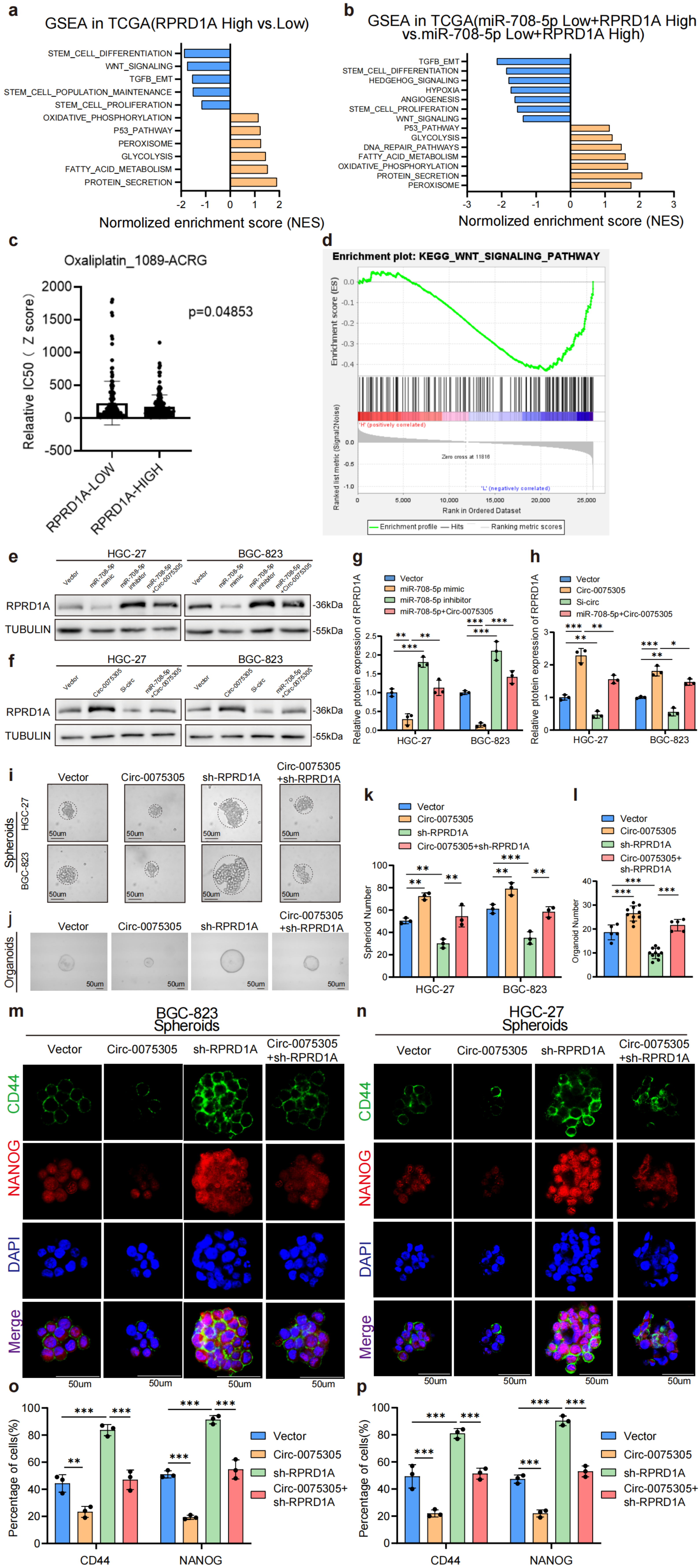

Supplementary Figure 11

a

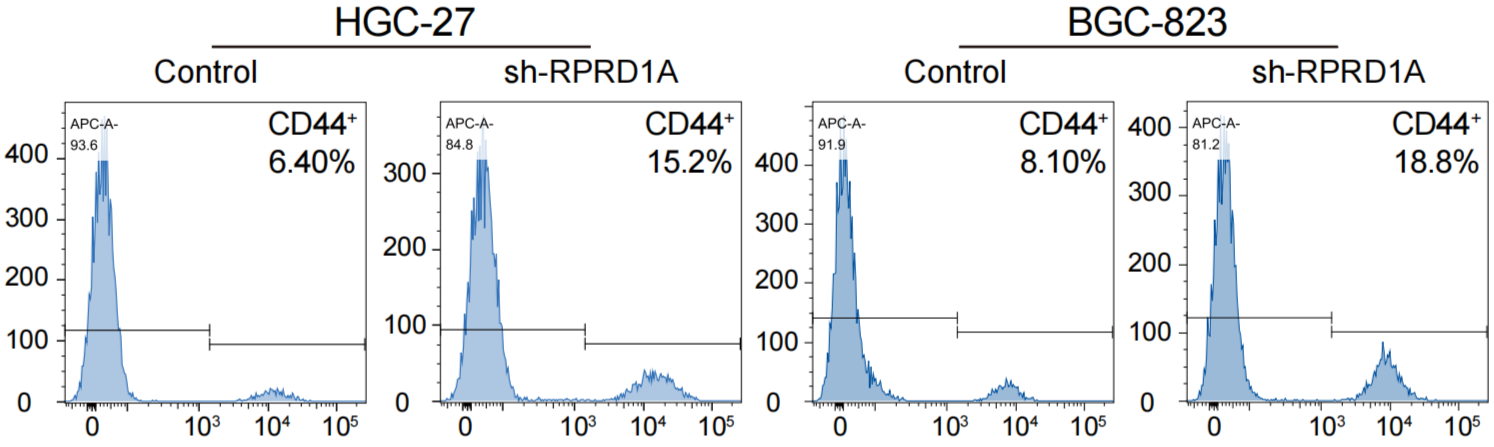

b

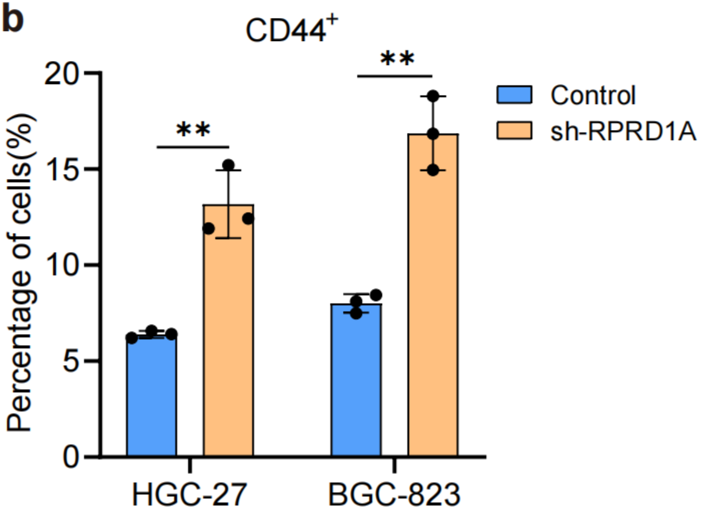

Supplementary Figure 12

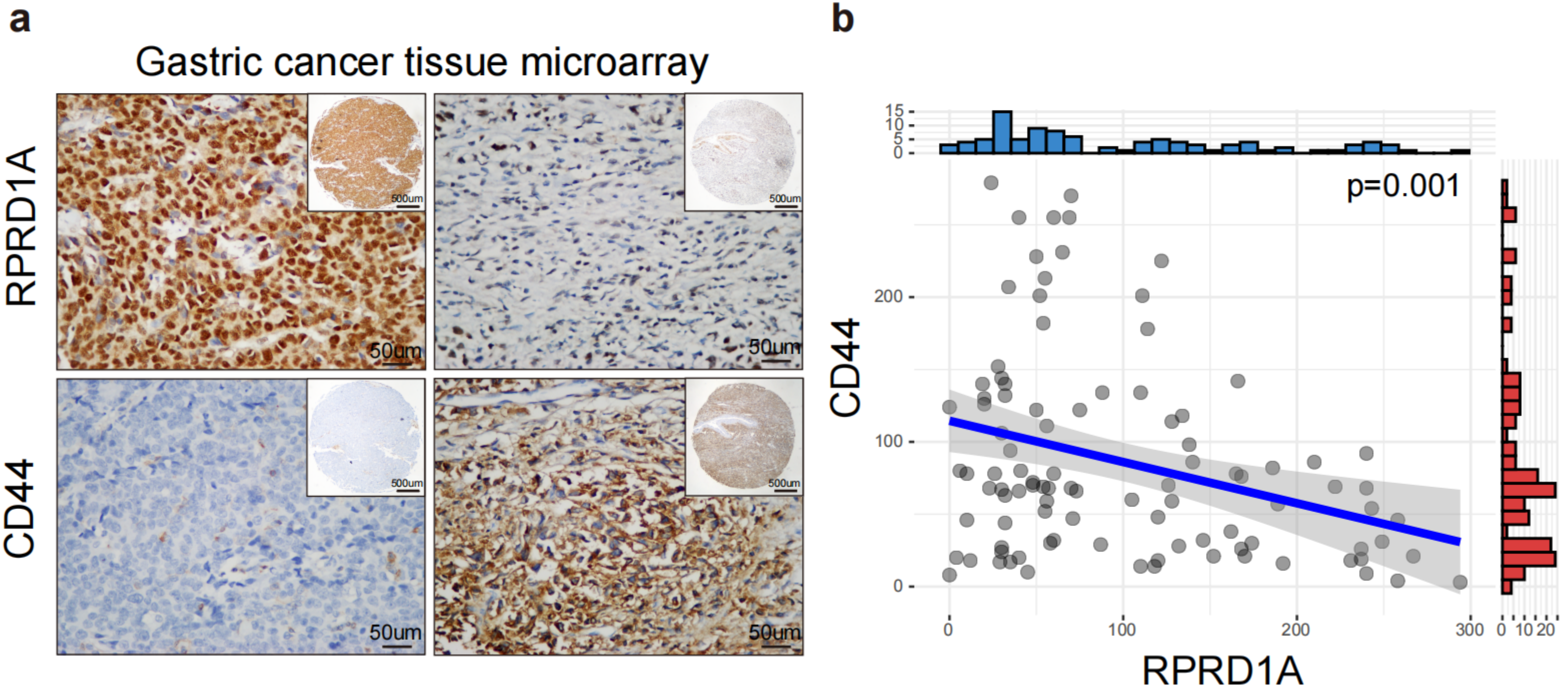

Supplementary Figure 13

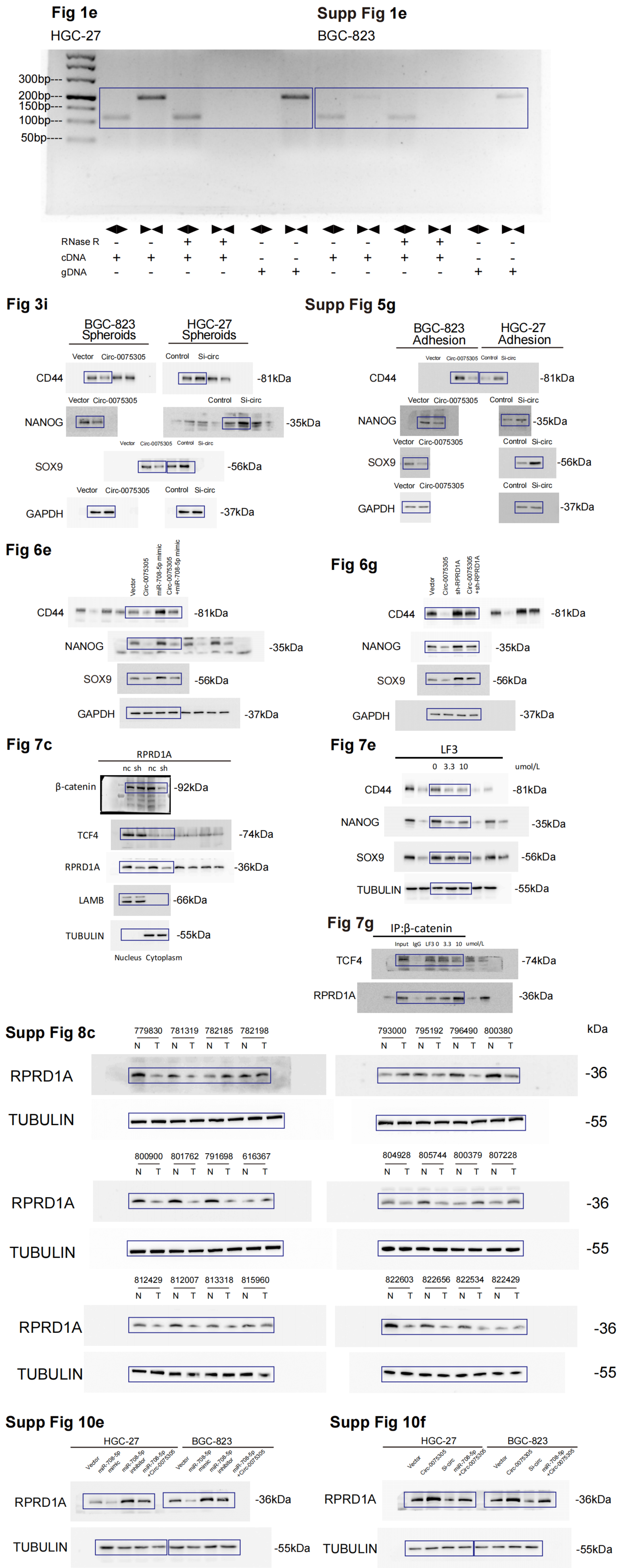

Supplementary Figure 14

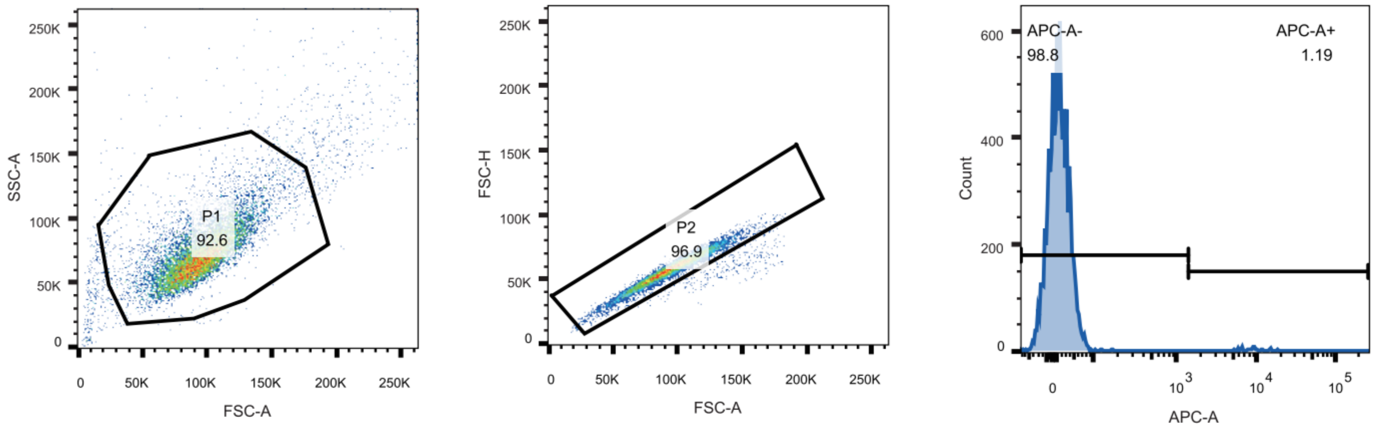

Supplement: Supplementary file 1 — Supplementary Information [file 42003_2024_6213_MOESM1_ESM.pdf]
