# Supplementary material for: Circ-0075305 hinders gastric cancer stem cells by indirectly disrupting TCF4–β-catenin complex and downregulation of SOX9
Source: Commun Biol. 2024 May 7;7:545. doi: 10.1038/s42003-024-06213-6 (PMC11076483; doi:10.1038/s42003-024-06213-6)
Supplement: Supplementary file 2 — Description of Additional Supplementary Files [file 42003_2024_6213_MOESM2_ESM.pdf]

Description of Additional Supplementary Files

File name: Supplementary Data 1

Description: The source data behind the graphs in the manuscript.
